# Supplementary material for: Descriptive epidemiology of the prevalence of adolescent active travel to school in Asia: a cross-sectional study from 31 countries
Source: BMJ Open. 2022 Apr 7;12(4):e057082. doi: 10.1136/bmjopen-2021-057082 (PMC8991057; doi:10.1136/bmjopen-2021-057082)
Supplement: Supplementary data [file bmjopen-2021-057082supp001.pdf]

Supplementary

Supplementary Table S1

Table S1. List of countries, year of surveys, and sample sizes from all 31 Asian countries

**Country**	**Year of survey**	**Sample size**
Eastern Mediterranean		
Afghanistan	2014	2028
Bahrain	2016	6928
Iraq	2012	1878
Jordan	2007	1852
Kuwait	2015	2983
Lebanon	2017	4451
Occupied Palestinian territory	2010	12224
Oman	2015	549
Pakistan	2009	4926
Qatar	2011	763
Syrian Arab Republic	2011	2932
United Arab Emirates	2016	5304
Yemen	2008	804
South East Asia		
Bangladesh	2014	2647
Bhutan	2016	7296
India	2007	6524
Indonesia	2015	10423
Maldives and Male	2009	1668
Myanmar	2016	2534
Nepal	2015	5700
Sri Lanka	2016	1618
Thailand	2015	5440
Timor-Leste	2015	2967
Western Pacific		

Brunei Darussalam	2014	2495
Cambodia	2013	3633
China	2003	7679
Lao People's Democratic Republic	2015	3527
Malaysia	2012	24711
Mongolia	2013	5252
Philippines	2015	7441
Vietnam	2013	3191

Supplementary Figure S1

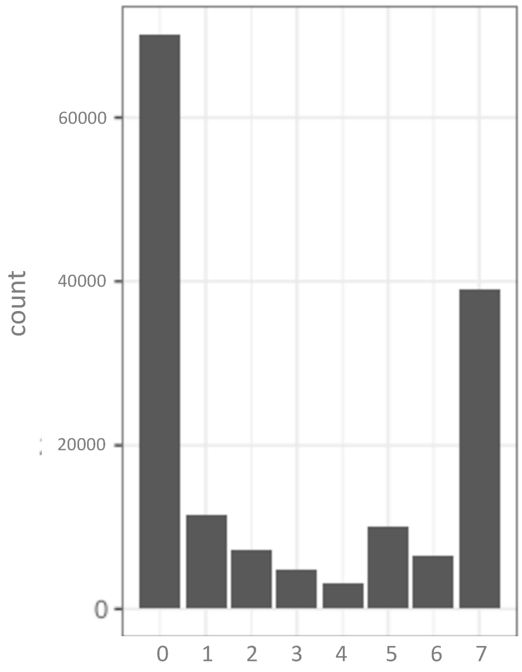

Figure S1. Count distribution of number of days of active travel to school (walking and cycling) in 31 Asian countries

Supplementary Figure S2

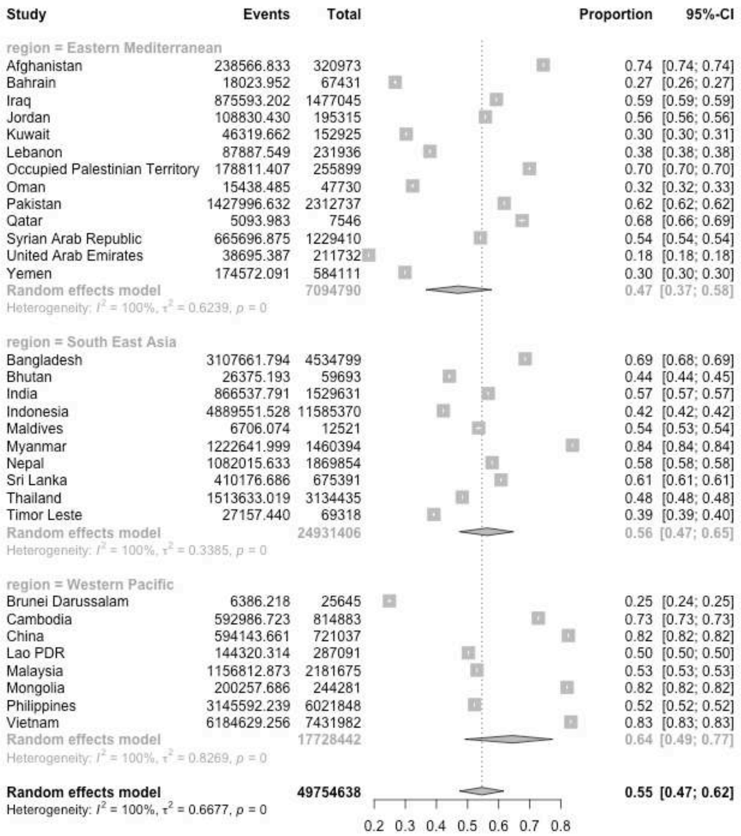

Figure S2. Meta-analysis of prevalence of active travel to school among adolescents in 31 Asian countries

Supplementary Table S2

Table S2. Characteristics comparison between adolescents included in the study, excluded from the study, and in the original dataset (unweighted)

	Complete dataset (n = 174,449)	Observations included in the study (n = 152,368) [a]	Observations with missing data (n = 22,081)	Observations missing weight and height data only (n =19,996)
Age group				
Younger	88,599 (50.8%)	75,655 (49.7%)	12,944 (58.6%)	11,873 (59.4%)
Older	84,957 (48.7%)	76,713 (50.3%)	8,244 (37.3%)	7,230 (36.2%)
NA	893 (0.5%)	-	893 (4.1%)	893 (4.4%)
Sex				
Male	83,090 (47.6%)	72,730 (47.7%)	10,360 (46.9%)	9,291 (46.5%)
Female	89,841 (51.5%)	79,638 (52.3%)	10,203 (46.2%)	9,187 (46.0%)
	1518 (0.9%)	-	1,518 (6.9%)	1,518 (7.5%)
ATS				
Yes	93,034 (53.3%)	82,291 (54.0%)	10,743 (48.7%)	10,743 (53.7%)
No	78,549 (45.0%)	70,077 (46.0%)	8,472 (38.4%)	8,472 (42.4%)
NA	2866 (1.7%)	-	2,866 (12.9%)	781 (3.9%)

[a] Observations with complete ATS, age, sex, weight and height were data included in the analysis.

Supplementary Table S3

Table S3. Meta-regression by year of survey

	**Prevalence**	**Age**	**Sex**	**BMI category**
τ^2 (estimated amount of residual heterogeneity)	0.34	0.023 (SE = 0.01)	0.076 (SE = 0.03)	0.02(SE = 0.01)
τ (square root of estimated tau squared value)	0.58	0.15	0.28	0.13
I^2 (residual heterogeneity / unaccounted variability)	99.99%	61.05%	89.01%	59.97%
H^2 (unaccounted variability / sampling variability)	89240.43	2.57	9.10	2.50
R^2 (amount of heterogeneity accounted for)	N/A	21.77%	7.13%	15.19%
Tests for residual heterogeneity	Wld (df = 19) = 762219.74 (p-value < .0001) LRT (df = 19) = 865578.68 (p-value < .0001)	QE (df = 19) = 43.62 (p-value = 0.01)	QE (df = 19) = 126.65 (p-value < .0001)	QE (df = 19) = 34.83 (p-value = 0.02)
Test of moderators (coefficients 2:12)	QM (df = 11) = 30.03 (p-value = 0.01)	QM (df = 11) = 15.12 (p-value = 0.18)	QM (df = 11) = 12.61 (p-value = 0.32)	QM (df = 11) = 11.36 (p-value = 0.41)

Df = data frame; LRT = likelihood ratio test statistic of the test for (residual) heterogeneity; QE = test statistic for the test of (residual) heterogeneity; QM = test statistic of the omnibus test of moderators; Wld = Wald-type test statistic of the test for residual heterogeneity
