# Supplementary material for: Descriptive epidemiology of the prevalence of adolescent active travel to school in Asia: a cross-sectional study from 31 countries
Source: BMJ Open. 2022 Apr 7;12(4):e057082. doi: 10.1136/bmjopen-2021-057082 (PMC8991057; doi:10.1136/bmjopen-2021-057082)
Supplement: Author's manuscript [file bmjopen-2021-057082.draft_revisions.pdf]

BMJ Open is committed to open peer review. As part of this commitment we make the peer review history of every article we publish publicly available.

When an article is published we post the peer reviewers' comments and the authors' responses online. We also post the versions of the paper that were used during peer review. These are the versions that the peer review comments apply to.

The versions of the paper that follow are the versions that were submitted during the peer review process. They are not the versions of record or the final published versions. They should not be cited or distributed as the published version of this manuscript.

BMJ Open is an open access journal and the full, final, typeset and author-corrected version of record of the manuscript is available on our site with no access controls, subscription charges or pay-per-view fees (http://bmjopen.bmj.com).

If you have any questions on BMJ Open's open peer review process please email info.bmjopen@bmj.com

BMJ Open

Descriptive epidemiology of adolescent active travel to school in Asia: a cross-sectional study from 31 countries

Journal:	*BMJ Open*
Manuscript ID	bmjopen-2021-057082
Article Type:	Original research
Date Submitted by the Author:	11-Sep-2021

Complete List of Authors:	Maulida, Rizka; University of Cambridge, MRC Epidemiology Unit; Universitas Indonesia, Faculty of Public Health<br>Ikeda, Erika; University of Cambridge, MRC Epidemiology Unit<br>Oni, T; University of Cambridge, MRC Epidemiology Unit<br>van Sluijs, Esther; University of Cambridge, MRC Epidemiology Unit

Keywords:	PUBLIC HEALTH, EPIDEMIOLOGY, Community child health < PAEDIATRICS

SCHOLARONE™
Manuscripts

I, the Submitting Author has the right to grant and does grant on behalf of all authors of the Work (as defined in the below author licence), an exclusive licence and/or a non-exclusive licence for contributions from authors who are: i) UK Crown employees; ii) where BMJ has agreed a CC-BY licence shall apply, and/or iii) in accordance with the terms applicable for US Federal Government officers or employees acting as part of their official duties; on a worldwide, perpetual, irrevocable, royalty-free basis to BMJ Publishing Group Ltd ("BMJ") its licensees and where the relevant Journal is co-owned by BMJ to the co-owners of the Journal, to publish the Work in this journal and any other BMJ products and to exploit all rights, as set out in our licence.

The Submitting Author accepts and understands that any supply made under these terms is made by BMJ to the Submitting Author unless you are acting as an employee on behalf of your employer or a postgraduate student of an affiliated institution which is paying any applicable article publishing charge ("APC") for Open Access articles. Where the Submitting Author wishes to make the Work available on an Open Access basis (and intends to pay the relevant APC), the terms of reuse of such Open Access shall be governed by a Creative Commons licence – details of these licences and which Creative Commons licence will apply to this Work are set out in our licence referred to above.

Other than as permitted in any relevant BMJ Author's Self Archiving Policies, I confirm this Work has not been accepted for publication elsewhere, is not being considered for publication elsewhere and does not duplicate material already published. I confirm all authors consent to publication of this Work and authorise the granting of this licence.

For peer review only

Descriptive epidemiology of adolescent active travel to school in Asia: a cross-sectional study from 31 countries

Rizka Maulida[1,2], Erika Ikeda[1], Tolu Oni[1], Esther MF van Sluijs[1]

1 MRC Epidemiology Unit, University of Cambridge, Cambridge, UK

2 Department of Epidemiology, Faculty of Public Health, Universitas Indonesia

Correspondence to Rizka Maulida: rizka.maulida@mrc-epid.cam.ac.uk

For peer review only

Abstract

Objectives: This study aimed to describe the epidemiology of adolescents' ATS in Asia. We aimed to examine the prevalence of adolescent ATS across 31 countries and territories in Asia, overall and by age group, sex and weight category.

Methods: This cross-sectional study used Global School-based student Health Survey (GSHS) data from 154,920 13-to-17-year-olds in 31 Asian countries. For each country, weighted prevalence of self-reported ATS (0 vs. 1-7 days) were calculated, and country-specific associations of ATS with age, sex and weight category were estimated using logistic regression. Summary prevalence and estimates were calculated using random-effects meta-analyses.

Results: Overall prevalence of adolescent ATS in Asia was 55%, ranging from 18% (United Arab Emirates) to 84% (Myanmar). There was limited sub-regional variation: 47% in the Eastern Mediterranean (EM), 56% in the South East Asia, and 64% in the Western Pacific. Being an older adolescent age 16 years and older (vs. younger age below 16 years: OR=1.08; 95%CI=1.00-1.16) was positively associated with ATS. This association was strongest in EM countries. Females (vs. males: 0.79; 0.71-0.89) and adolescents with overweight/obesity (vs. normal weight: 0.92; 0.86-0.99) were less likely to use ATS. Association with sex was strongest in EM countries. Heterogeneity was considerable in all meta-analyses.

Conclusion: The prevalence of adolescent ATS in Asia varies substantially. Overall, older and male adolescents, and adolescents with normal and below normal weight category are more likely to actively travel to school. However, the main contributor to differences in ATS between and within regions remain unknown. Although there is substantial scope for improving ATS rates in Asia, any policy action should be cognisant of local built, social, and natural environmental contexts that may influence active travel behaviour.

For peer review only

Article Summary

Strengths and limitations of this study

- This study pooled comparable estimates on active travel to school from 154,920 adolescents from 31 countries in Asia
- Data were collected using standardised sampling and data collection methods, enabling comparison across countries and sub-regions
- Data were from low- and middle-income countries thus the extent to which these findings are generalisable to other, particularly high-income, countries in Asia is unclear
- Data collection was conducted in schools, and thus the conclusions drawn here are only relevant to adolescents in school

For peer review only

Introduction

Globally, non-communicable diseases (NCDs), notably cancers, cardiovascular diseases, type 2 diabetes and chronic respiratory illness, are responsible for about 40 million deaths each year (1). NCDs have been associated with various modifiable behavioural risk factors including tobacco use, physical inactivity, unhealthy diet and the harmful use of alcohol (1, 2). The risk of developing NCDs can be lowered by avoiding these unhealthy behaviours, for example, by being physically active (3, 4). Physical inactivity-related diseases mostly manifest in adulthood but their development starts in childhood and adolescence (5).

Studies have reported that health benefits gained from physical activity in adolescence, defined as age 10-19 years (6), are carried forward into adulthood (7-9). Despite these benefits, global research on physical activity using self-reported questionnaires have shown that more than 80% of adolescents are not meeting international guidelines of engaging in 60 minutes of moderate-to-vigorous physical activity on average per day (10). Moreover, evidence suggests that physical activity starts to decline from childhood and continues to decline into adulthood (11-13). By contrast, a continuous high level of physical activity in general (14), and maintained active travel, for example walking or cycling, in particular (15), throughout adolescence significantly predicts a high level of adult physical activity. Therefore, as adolescence is a key period for establishing active living habits, promoting active travel in this period is important to increase overall physical activity among adolescents. Given that the period of adolescence is also the age range for school attendance, active travel to school (ATS) could be an important focus for interventions to increase active travel in this age group.

A systematic review among school children demonstrated that those who actively travelled to school were more likely to be physically active overall (16). A single walk to school has been estimated to contribute between 16-18% of younger adolescents' daily moderate-to-vigorous physical activity (17). However, data on the prevalence of adolescent ATS are scarce. A study using data from the Global Matrix 3.0 showed that half of children and adolescents actively travelled to and from places, where these trips are mainly trips to and/or from school (18). However, this study was unable to generate a pooled estimate of the global prevalence of active travel due to different methodologies used in the countries studied. More recently, a study using comparable data of ATS in 63 low- and middle-income countries

For peer review only - http://bmjopen.bmj.com/site/about/guidelines.xhtml

revealed a prevalence of 56.1% (19). Using a mix of 27 Asian and Pacific countries, another study reported a prevalence of 42.1% (20). To date, no studies have solely focused their analyses on Asia (18-20).

Asia is the largest and most populous continent, with a population of 4.6 billion (21), where many countries are emerging economies. Rapid urbanisation and dynamically changing built environments in the region also pose a challenge for active travel. These built environments also pose a broader risk to planetary health, the interaction between human health and the environments we depend on (22). For example, the cities with the worst air pollution levels globally are within greater Asia (23), and air pollution has negative impacts both on the climate, as well as health outcomes and risks associated with physical activity outdoors (24). To better understand the potential for built environment interventions to influence ATS behaviours in adolescents, it is vital to have a better understanding of the pre-existing prevalence in this population group and region. But the paucity of evidence on the overall prevalence of ATS in Asia poses a challenge for evidence-informed policies to promote ATS.

Existing evidence from Asia on ATS (25-30) suggests that boys are more likely to use active modes of travel compared to girls, and students living in less wealthy areas or studying in less wealthy areas are more likely to use active travel than their counterparts. In addition, ATS is positively associated with (perceived- and objectively-measured) shorter distance to school, favourable built environments (e.g., larger street block size and tree cover), and higher social interaction (26-30). However, these studies had relatively small sample sizes (between 330 and 1518 participants) and have been conducted in a limited number of Asian countries, mostly in East and Southeast Asia.

The goal of this study was to examine the descriptive epidemiology of active travel to school among adolescents in Asian countries. We aimed to examine the prevalence of adolescent ATS across 31 countries and territories in Asia, overall and by age group, sex and weight category.

For peer review only

Methods

The reporting of this paper follows the Strengthening the Reporting of OBservational studies in Epidemiology (STROBE) guideline (31).

Data source

This study used data from the Global School-based Student Health Survey (GSHS), developed by the World Health Organization (WHO) and the Center for Disease Control and Prevention (CDC) in collaboration with UNICEF, UNESCO, and UNAIDS. The GSHS is a school-based survey conducted mostly among students aged 13–17 years, but some countries also include those aged 11–12 and 18 years (32). Detailed information about the GSHS can be found on WHO and CDC websites (33, 34).

GSHS used a two-staged cluster sampling design to obtain a nationally representative sample of the adolescents. In the first stage, schools were randomly selected using the probability proportional to size sampling. In the second stage, classes were randomly selected in the selected schools, with varied number of classes depending on the school-size. All students in the selected classes were eligible to participate in the survey, and the participation was anonymous and voluntary.

The GSHS surveys have been conducted in 185 mostly low- and middle-income countries globally from 2003 to 2017. The survey consists of 10 core modules on adolescent health behaviours including physical activity and nutrition and protective factors, along with other optional modules. All GSHS datasets are freely available on the WHO's website: https://www.who.int/teams/noncommunicable-diseases/surveillance/systems-tools/global-school-based-student-health-survey/.

Study design and ethics

This cross-sectional study used GSHS data from 31 Asian countries, as defined by the United Nations' Statistics Division of geographic regions (35). All 31 countries assessed ATS using GSHS surveys. The GSHS survey in each country was approved by the Ministry of Education, Ministry of Health, or other institution in charge of the survey and underwent ethical clearance. Only those adolescents and their parents who provided written or verbal consent participated. As the current study used retrospective, de-identified, publicly available data, ethics approval was not required for this secondary analysis.

For peer review only - http://bmjopen.bmj.com/site/about/guidelines.xhtml

Data management

Data management was done using RStudio version 1.4. Prior to WHO data publication, out of range edits, multiple response edits, logical consistency edits, height and weight edits, variable edits, and record-level edits were performed on all GSHS datasets (32). All 31 country datasets were drawn from the most recent GSHS survey in each country, except for Maldives and Oman which did not have national datasets for physical activity components in their most recent datasets. All 31 datasets were checked for missing values and observations with missing values were dropped.

Study variables

The outcome variable, active travel to school, was self-reported using the question "During the past seven days, on how many days did you walk or ride a bicycle to or from school?" with eight responses ranging from 0-7 days. In this study, travel to school was dichotomised into *passive* (0 days) and *active* (1-7 days) travel. This categorisation was chosen based on the distribution of ATS across the dataset which showed high counts of 0 days, small counts of 1-4 days, and higher counts of 5-7 days.

Independent variables included age, sex, and weight category. Age (in years) and sex (male or female) were self-reported. For analysis, age was categorised into two groups: *younger* (under 16 years old) and *older* (16 years old and over) adolescents. This cut-off point was as per standard GSHS reporting (36). Sex was expressed as '1' for *male*, and '2' for *female*. Participants' height and weight were measured by survey staff before survey administration. Measurements were written by staff on slips of paper and given to each participant to be entered into their GSHS answer sheet. Weight category was generated based on the WHO categorisation of Body Mass Index (BMI)-for-age among 5–19-year-old-children, and then categorised into: *underweight and normal* or *above normal* ('overweight' and 'obese') (37).

Statistical analysis

All statistical analyses were conducted using RStudio version 1.4. Only participants with complete data were included in the analyses. The 31 countries were categorised into three sub-regions: Eastern Mediterranean (which mostly consists of Middle Eastern countries); South East Asia (which consists of countries in the South and some countries in the Southeast Asia region); and Western Pacific (which consists of the rest of the countries in the Southeast Asia region along with countries in East and Central Asia). This categorisation was based on

For peer review only

cultural and climate similarities shared by these countries, as well as WHO regional offices (38). To adjust for nonresponse and distribution of adolescents by cluster, a sampling (survey) weight was applied to each adolescent. These weights were generated by accounting the size of cluster the where the adolescent was sampled. Percentages and association estimates were also calculated under these weights to be representative of all students in each cluster. Logistic regression modelling was used to estimate each country's weighted associations of ATS, as independent variable, with age group, sex and weight category, as dependent variables. Younger (<16 years old) adolescents, male adolescents, and adolescents with underweight or normal weight category were used as the reference category. The weighted percentages and estimates (i.e., odds ratio (OR)) were pooled using random-effects meta-analysis to obtain the overall prevalence and estimates for the 31 countries. I^2 was used to determine the importance of heterogeneity in each meta-analysis (0-40%: might not be important, 30-60%: may represent moderate heterogeneity, 50-90%: may represent substantial heterogeneity, 75-100%: considerable heterogeneity) (39). We stratified the meta-analysis by sub-region to establish potential regional differences. We also performed meta-regression to check the potential impact of year of survey variability. Year of survey, along with sample size, for each country is shown on Supplementary Table S1.

For peer review only

For peer review only - http://bmjopen.bmj.com/site/about/guidelines.xhtml

Results

Of the original 176,311 adolescents surveyed from the 31 countries, there were 154,920 (87.9%) adolescents aged 11-18 years with complete data included in the analysis. Participants were excluded due to missing values in one or more variables: n=893 for age, n=1,518 for sex, n=19,996 for weight and height, and n=2,866 for travel to school. Table 1 shows the characteristics of adolescents included in the analyses, stratified by country. The proportion of younger adolescents varied across countries: from 50% in Thailand to only 15% in Lao People's Democratic Republic. Across all countries, percentages of male participants were relatively equal, ranging between 45% and 50%. With respect to weight category, Kuwait had the highest percentage of adolescents with overweight or obesity (51%), and Vietnam (6.2%) and Timor Leste (6.2%) had the lowest.

Overall active travel to school prevalence

The overall prevalence of adolescent ATS in Asia was 55.0% (see forest plot in Supplementary Figure S1). Figures 1 and 2 show a map and a forest plot of the weighted prevalence of ATS in the 31 Asian countries, respectively. The forest plot is stratified by sub-region and sorted from highest to lowest prevalence. In the Eastern Mediterranean, the ATS prevalence ranged from 18% (United Arab Emirates) to 74.0% (Afghanistan), with a median value of 54%. In the South East Asia, the prevalence of ATS ranged from 39% (Timor Leste) to 84% (Myanmar), with a median value of 56%. In the Western Pacific, ATS prevalence ranged from 25.0% (Brunei Darussalam) to 82.0% (China), with a median value of 63.0%.

Active travel to school association with age, sex, and weight category

Overall, older adolescents were 8% more likely to actively travel to school compared to younger adolescents (OR: 1.08; 95% CI: 1.00, 1.16) (Figure 3a). This association was strongest in the Eastern Mediterranean countries (OR: 1.13; 95% CI: 1.04, 1.23), whereas no association was observed in the other two Asian sub-regions. Figure 3b shows that female adolescents were 21% less likely to actively travel to school than male adolescents (OR: 0.79; 95% CI: 0.71, 0.89). Stratified analyses showed that this association was strongest in Eastern Mediterranean countries (OR: 0.67; 95% CI: 0.55, 0.81), and then in South-East Asia countries (OR: 0.79; 95% CI: 0.69, 0.90). No association was observed in Western Pacific countries. Lastly,

For peer review only - http://bmjopen.bmj.com/site/about/guidelines.xhtml

adolescents with overweight and obesity were 8% less likely to actively travel to school than those with underweight or normal (OR: 0.92; 95% CI: 0.86, 0.99) (Figure 3c) weight categories. However, stratified analyses did not show any statistically significant associations by sub-region.

Heterogeneity

Overall heterogeneity (I^2) was considerable in all three meta-analyses (age group: 64%, sex: 87%, and weight category: 51%) (Figure 3). Some region-specific variations were observed, with only the association with sex showing considerable heterogeneity (83%) in the Eastern Mediterranean; whereas considerable heterogeneity was observed across all meta-analyses in the Western Pacific. Meta-regression shows that year of survey accounted for some of this heterogeneity (Supplementary Table S2).

For peer review only

For peer review only - http://bmjopen.bmj.com/site/about/guidelines.xhtml

Discussions

Main findings

This study showed that 55% of Asian adolescents walk or cycle to school at least once per week. This prevalence varied substantially by country, with estimates varying from 18% (United Arab Emirates) to 84% (Myanmar). However, regional variation was limited with 54% ATS prevalence noted in the Eastern Mediterranean, 55.5% in the South East Asia, and 63% in the Western Pacific. Overall, older adolescents, male adolescents, and adolescents with underweight/normal weight were more likely to use active modes than their counter parts. However, these associations varied across countries, suggesting that there may be substantial country-level variation in determinants of ATS.

Active travel to school prevalence

The prevalence observed in the current study (55%) is broadly similar to that reported in previous studies from various countries and regions (18-20). However, this is lower than global estimates in a recent review, which reported that 62% of boys and 54% girls actively travel to school (40). Due to methodological differences between studies, our study's prevalence can only be compared directly to prevalence obtained from other GSHS studies (19, 20) and comparison to other studies should be done with caution.

Despite limited regional variation, the Eastern Mediterranean had the lowest prevalence of adolescent ATS, which might reflect the high prevalence of physical inactivity in the general adolescent population in Eastern Mediterranean countries (10). High physical inactivity, which includes ATS, may be due to the climate in the region. Evidence from research on adults in Saudi Arabia showed that half of participants were hesitant to walk daily due to the hot weather during the summer (41). However, this evidence did not show that the hesitancy changed with changing seasons. Furthermore, other studies of children and adolescents conducted in European countries have shown that temperature, precipitation, and wind speed were associated with various domains of physical activity, including ATS (42-44).

For peer review only

Age

Similar to our findings, a study in Hong Kong reported that older adolescents were more likely to actively travel to school (30). However, studies in other Asian countries have not reported differences in active travel between age groups (26-29). Coincidentally, most of these previous studies come from Asian countries belonging to the Western Pacific, where we also found no difference of ATS prevalence between younger and older adolescents. In our study, difference in ATS prevalence by age group was also not noted in South East Asia countries but only present in Eastern Mediterranean countries. A plausible explanation could be that the age when adolescents are permitted to travel independently differ by countries, perhaps due to varying perceptions of risks of injury or violence. For instance, age for independent mobility in South East Asia and Western Pacific countries may be lower than our study's age categorisation cut-off point. However, to our knowledge, no studies have explored this for the Asian region.

Sex

Findings from both our study and previous research using the GSHS and other global datasets demonstrated that female adolescents in Asia are less likely to actively travel to school than male adolescents (25, 27, 45, 46). Similarly, among United States of America and European adolescents, female adolescents accumulated lower overall physical activity compare to male adolescents (40). In general, female adolescents in Asia are more restricted in active travel due to cultural norms (47). For example, a study among Indian adults showed that cycling is deemed appropriate for men but improper and unacceptable for women (48). Another study from Saudi Arabia also reported that women's presence on the streets is limited because urban streets are considered a domain for men (49). These cultural expectations may act as a barrier to increasing the prevalence of ATS amongst adolescent females in Asia.

Weight category

Although adolescents with above normal weight were less likely to actively travel to school, this association was not observed in the sub-region stratified analyses. Strong associations were only present in Cambodia and Vietnam, which is similar to findings from a Dutch population study where BMI was found to negatively influence later levels of active

For peer review only - http://bmjopen.bmj.com/site/about/guidelines.xhtml

travel (50). The results of our study suggest that weight category may not a significant determinant of ATS in most Asian countries. A plausible explanation for this finding could be that those adolescents with above normal weight are equally motivated to engage in active travel in an effort to obtain healthier weight. However, the association of weight and active travel may also be bi-directional. For example, a Danish adolescent study reported that cycling to school was associated with lower BMI (51).

Strengths and limitations

This study pooled estimates from 154,920 adolescents from 31 countries in Asia. Sub-region and age-, sex- and weight-specific estimates were also analysed. Data were collected using standardised sampling and data collection methods, enabling comparison across countries and sub-regions. However, the prevalence of ATS was obtained from one single self-reported question. Participating countries in the GSHS were also mostly those categorised as low- and middle-income countries in Asia. As a result, the extent to which these findings are generalisable to other, particularly high-income, countries in Asia is unclear. This study also dichotomised both dependent and independent variables for meta-analysis purposes, which may have contributed to biases in the results, such as loss of information about individual differences and loss of effect (52). Data on response rates from each survey and characteristics of non-respondents were not available, but we used the study weights provided to obtain representative estimates. Data collection was conducted in schools, and thus the conclusions drawn here are only relevant to adolescents in school, however out of school adolescents account for approximately 7% in Asia as compared to global percentage of 15% (53). The GSHS also mainly focuses on adolescents aged 13-17 years old, and the proportion of 11-12 years old were much smaller. Therefore, the evidence from adolescents in this younger age group may be underrepresented. Lastly, the GSHS data set does not contain information on individual-level socio-economic position, which may be an important determinant of ATS.

Implications

There is substantial scope for improving ATS prevalence in Asian countries. The prevalence of ATS varies between and within the three Asian sub-regions. Many of associations with individual factors (i.e., age group, sex and weight category) also differ across

For peer review only - http://bmjopen.bmj.com/site/about/guidelines.xhtml

the sub-regions. Future research is needed to explore why these variations occur among these countries, and how these findings may inform future policies on ATS. Any such policy actions will need to be contextual, cognisant of local built, social, and natural environmental contexts that could influence ATS. Further studies are therefore needed to gather evidence on the roles that environmental factors, such as exposure to air pollution, high density traffic, walkability, and safety, play in influencing ATS in Asia (54). Such studies could build on child-friendly city initiatives (55) to generate evidence to inform strategies to equitably improve adolescent ATS across the region. Furthermore, there is a need to explore gendered cultural norms in the context of Asian countries, to ensure interventions designed to encourage ATS do not exclude female adolescents.

Conclusions

The overall prevalence of adolescent ATS in Asia was 55%. The prevalence was lowest in United Arab Emirates and highest in Myanmar. Overall, older adolescents, male adolescents, and adolescents with underweight and normal weight were more likely to actively travel to school than their counterparts. Although age, sex, and weight status were associated with the prevalence of ATS to varying degrees in the Eastern Mediterranean, South East Asia, and Western Pacific regions, the main driver of variation within and between Asian countries remains unknown. This highlights the importance of contextually tailored interventions in future efforts to improve adolescent ATS. Further investigation to identify other potential factors which account for differences in adolescent ATS prevalence across Asian countries is therefore needed to inform policy and practice.

For peer review only - http://bmjopen.bmj.com/site/about/guidelines.xhtml

Declarations

Funding

RM's doctoral study is supported by Indonesia Endowment Fund for Education (201908220815174). EMFvS and EI are supported by the Medical Research Council [grant number MC_UU_00006/5]. TO is supported by the National Institute for Health Research (NIHR) (16/137/34) using UK aid from the UK Government to support global health research.

Contributors

RM designed the study with the supervision of EMFvS and TO. RM collected and managed the data assisted by EI. RM conducted the statistical analysis with guidance from EMFvS and EI. RM drafted the paper. All authors reviewed the results, edited the manuscript and agreed on the final version of the manuscript.

Ethic statement

In each individual country, the GSHS was approved by the Ministry of Education, Ministry of Health, or other institution in charge of the survey. Ethical approval was also obtained in all 31 individual countries. No reference numbers for these ethical approvals are available. Only those adolescents and their parents who provided written or verbal consent participated. As the current study used retrospective, de-identified, publicly available data, no separate ethics approval was required for the analysis of the data.

Competing interests

None declared.

Data sharing statement

Original GSHS datasets are available publicly online at https://www.who.int/teams/noncommunicable-diseases/surveillance/systems-tools/global-school-based-student-health-survey. Processed datasets used for this study and R codes used for data management and data analysis are available by emailing Rizka Maulida at rizka.maulida@mrc-epid.cam.ac.uk

For peer review only

Disclaimer

The views expressed in this publication are those of the authors and not necessarily those of the funders. The funders had no role in the study.

Map disclaimer

The depiction of boundaries on the map(s) in this article does not imply the expression of any opinion whatsoever on the part of BMJ (or any member of its group) concerning the legal status of any country, territory, jurisdiction or area or of its authorities. The map(s) are provided without any warranty of any kind, either express or implied.

Acknowledgements

The authors would like to thank Stephen Sharp of MRC Epidemiology Unit, University of Cambridge and Ihsan Fadilah of Eijkman-Oxford Clinical Research Unit, Jakarta, for the support on data analysis, and Bimandra Djaafara of Imperial College, London, for the assistance on map creation.

Patient and public involvement

Patients or the public WERE NOT involved in the design, or conduct, or reporting, or dissemination plans of our research.

Twitter

Rizka Maulida @rizkamaulida13, Erika Ikeda @DrErikaIkeda, Tolu Oni @DrTolullah and Esther MF van Sluijs @EvanSluijs

For peer review only

Table 1. Characteristic of adolescents included in analyses from all 31 Asian countries

Country	Population[a]	Age category[b]		Sex		Weight category[c]	
		Younger	Older	Male	Female	Underweight and normal	Above normal
Eastern Mediterranean							
Afghanistan	320,973	34%	66%	54%	46%	83%	17%
Bahrain	67,431	54%	46%	50%	50%	58%	42%
Iraq	1,477,045	51%	49%	57%	43%	73%	27%
Jordan	195,315	37%	63%	52%	48%	76%	24%
Kuwait	152,925	36%	64%	51%	49%	49%	51%
Lebanon	231,936	44%	56%	46%	54%	72%	28%
Occupied Palestinian territory	255,899	70%	30%	49%	51%	74%	26%
Oman	47,730	30%	70%	47%	53%	76%	24%
Pakistan	2,312,737	61%	39%	61%	39%	92%	8.1%
Qatar	7,546	81%	19%	52%	48%	50%	50%
Syrian Arab Republic	1,229,410	75%	25%	51%	49%	73%	27%
United Arab Emirates	211,732	38%	62%	49%	51%	59%	41%
Yemen	584,111	57%	43%	64%	36%	88%	12%
South East Asia							
Bangladesh	4,534,799	64%	36%	64%	36%	89%	11%

For peer review only - http://bmjopen.bmj.com/site/about/guidelines.xhtml

Bhutan	59,693	29%	71%	48%	52%	87%	13%
India	1,529,631	65%	35%	58%	42%	88%	12%
Indonesia	11,585,370	67%	33%	48%	52%	82%	18%
Maldives and Male	12,521	35%	65%	49%	51%	88%	12%
Myanmar	1,460,394	65%	35%	45%	55%	92%	8.4%
Nepal	1,869,854	54%	46%	48%	52%	92%	8.0%
Sri Lanka	675,391	39%	61%	46%	54%	85%	15%
Thailand	3,134,435	50%	50%	45%	55%	81%	19%
Timor-Leste	69,318	22%	78%	50%	50%	94%	6.2%
Western Pacific							
Brunei Darussalam	25,645	49%	51%	49%	51%	63%	37%
Cambodia	814,883	31%	69%	52%	48%	96%	3.8%
China	721,037	73%	27%	51%	49%	82%	18%
Lao People's Democratic Republic	287,091	15%	85%	53%	47%	88%	12%
Malaysia	2,181,675	41%	59%	50%	50%	76%	24%
Mongolia	244,281	49%	51%	48%	52%	88%	12%
Philippines	6,021,848	49%	51%	49%	51%	90%	10%
Vietnam	7,431,982	21%	79%	47%	53%	94%	6.2%

[a] population represented by the sample; [b] age was categorised into younger (< 16 years old), older (≥ 16); [c] weight category was dichotomised by WHO categorisation of Body Mass Index (BMI)-for-age among 5-19-year-old children, where above normal included overweight and obese categories.

For peer review only - http://bmjopen.bmj.com/site/about/guidelines.xhtml

References

1. WHO. Non communicable diseases 2018 [Available from: http://www.who.int/news-room/fact-sheets/detail/noncommunicable-diseases.
2. Collaborators GBDRF. Global, regional, and national comparative risk assessment of 79 behavioural, environmental and occupational, and metabolic risks or clusters of risks, 1990-2015: a systematic analysis for the Global Burden of Disease Study 2015. Lancet. 2016;388(10053):1659-724.
3. Beaglehole R, Yach D. Globalisation and the prevention and control of non-communicable disease: the neglected chronic diseases of adults. Lancet. 2003;362(9387):903-8.
4. Collaborators GBDRF. Global, regional, and national comparative risk assessment of 84 behavioural, environmental and occupational, and metabolic risks or clusters of risks, 1990-2016: a systematic analysis for the Global Burden of Disease Study 2016. Lancet. 2017;390(10100):1345-422.
5. Parsons TJ, Power C, Logan S, Summerbell CD. Childhood predictors of adult obesity: a systematic review. Int J Obes Relat Metab Disord. 1999;23 Suppl 8:S1-107.
6. Organization WH. Adolescence: a period needing special attention - recognizing-adolescence. 2021.
7. Sallis JF, Owen N. Physical Activity and Behavioral Medicine: SAGE Publications; 1999.
8. WHO. Global Recommendations on Physical Activity for Health. Global Recommendations on Physical Activity for Health. WHO Guidelines Approved by the Guidelines Review Committee. Geneva2010.
9. Dick B, Ferguson BJ. Health for the world's adolescents: a second chance in the second decade. J Adolesc Health. 2015;56(1):3-6.
10. Guthold R, Stevens GA, Riley LM, Bull FC. Global trends in insufficient physical activity among adolescents: a pooled analysis of 298 population-based surveys with 1.6 million participants. The Lancet Child & adolescent health. 2019.
11. Farooq MA, Parkinson KN, Adamson AJ, Pearce MS, Reilly JK, Hughes AR, et al. Timing of the decline in physical activity in childhood and adolescence: Gateshead Millennium Cohort Study. British journal of sports medicine. 2018;52(15):1002-6.
12. Corder K, Winpenny E, Love R, Brown HE, White M, Sluijs EV. Change in physical activity from adolescence to early adulthood: a systematic review and meta-analysis of longitudinal cohort studies. British journal of sports medicine. 2019;53(8):496-503.
13. Winpenny EM, Smith M, Penney T, Foubister C, Guagliano JM, Love R, et al. Changes in physical activity, diet, and body weight across the education and employment transitions of early adulthood: A systematic review and meta-analysis. Obes Rev. 2020;21(4):e12962.
14. Telama R, Yang X, Viikari J, Valimaki I, Wanne O, Raitakari O. Physical activity from childhood to adulthood: a 21-year tracking study. Am J Prev Med. 2005;28(3):267-73.
15. Yang X, Telama R, Hirvensalo M, Tammelin T, Viikari JS, Raitakari OT. Active commuting from youth to adulthood and as a predictor of physical activity in early midlife: the young Finns study. Preventive Medicine. 2014;59:5-11.
16. Larouche R, Saunders TJ, Faulkner G, Colley R, Tremblay M. Associations between active school transport and physical activity, body composition, and cardiovascular fitness: a systematic review of 68 studies. Journal of physical activity & health. 2014;11(1):206-27.

For peer review only - http://bmjopen.bmj.com/site/about/guidelines.xhtml

17. Southward EF, Page AS, Wheeler BW, Cooper AR. Contribution of the school journey to daily physical activity in children aged 11-12 years. Am J Prev Med. 2012;43(2):201-4.
18. Gonzalez SA, Aubert S, Barnes JD, Larouche R, Tremblay MS. Profiles of Active Transportation among Children and Adolescents in the Global Matrix 3.0 Initiative: A 49-Country Comparison. Int J Environ Res Public Health. 2020;17(16).
19. Peralta M, Henriques-Neto D, Bordado J, Loureiro N, Diz S, Marques A. Active Commuting to School and Physical Activity Levels among 11 to 16 Year-Old Adolescents from 63 Low- and Middle-Income Countries. Int J Environ Res Public Health. 2020;17(4).
20. Uddin R, Mandic S, Khan A. Active commuting to and from school among 106,605 adolescents in 27 Asia-Pacific countries. Journal of transport & health. 2019;15:100637.
21. United Nations DoEaSA, Population Division. World Population Prospects 2019 2019 [Available from: https://population.un.org/wpp/.
22. Prior JH, Connon IL, McIntyre E, Adams J, Capon A, Kent J, et al. Built environment interventions for human and planetary health: integrating health in climate change adaptation and mitigation. Public Health Res Pract. 2018;28(4).
23. IQAir. 2020 World Air Quality Report. 2021.
24. Manisalidis I, Stavropoulou E, Stavropoulos A, Bezirtzoglou E. Environmental and Health Impacts of Air Pollution: A Review. Frontiers in public health. 2020;8:14.
25. Tudor-Locke C, Ainsworth BE, Adair LS, Popkin BM. Objective physical activity of filipino youth stratified for commuting mode to school. Med Sci Sports Exerc. 2003;35(3):465-71.
26. Lin J-J, Chang H-T. Built Environment Effects on Children's School Travel in Taipai: Independence and Travel Mode. Urban Studies. 2009;47(4):867-89.
27. Trang NH, Hong TK, Dibley MJ. Active commuting to school among adolescents in Ho Chi Minh City, Vietnam: change and predictors in a longitudinal study, 2004 to 2009. Am J Prev Med. 2012;42(2):120-8.
28. Leung A, Le TPL. Factors associated with adolescent active travel: A perceptive and mobility culture approach – Insights from Ho Chi Minh City, Vietnam. Transportation Research Part A: Policy and Practice. 2019;123:54-67.
29. Barnett A, Akram M, Sit CH, Mellecker R, Carver A, Cerin E. Predictors of healthier and more sustainable school travel mode profiles among Hong Kong adolescents. Int J Behav Nutr Phys Act. 2019;16(1):48.
30. Leung KYK, Loo BPY. Determinants of children's active travel to school: A case study in Hong Kong. Travel Behaviour and Society. 2020;21:79-89.
31. STROBE Statement—Checklist of items that should be included in reports of cross-sectional studies 2009 [Available from: https://www.strobe-statement.org/fileadmin/Strobe/uploads/checklists/STROBE_checklist_v4_cross-sectional.pdf.
32. Control CfD, Prevention, Organization WH. GSHS data user's guide. 2015.
33. CDC Global School-based Student Health Survey (GSHS) 2019 [updated 2019-05-23T02:57:19Z/. Available from: https://www.cdc.gov/gshs/index.htm.
34. Larsen K, Buliung R, Faulkner G. Safety and school travel: How does the environment along the route relate to safety and mode choice? Transp Res Rec [Internet]. 2013 Jan 1 [cited 2016 Nov 1]; 2327(1):[9-18 pp.]. Available from: https://journals.sagepub.com/doi/10.3141/2327-02.
35. Division UNS. Methodology: Standard country or area codes for statistical use (M49) 2020 [Available from: https://unstats.un.org/unsd/methodology/m49/.

For peer review only - http://bmjopen.bmj.com/site/about/guidelines.xhtml

36. Regions - CDC Global School-based Student Health Survey (GSHS) 2019 [updated 2019-08-09T01:12:12Z/. Available from: https://www.cdc.gov/gshs/countries/index.htm.
37. Organization WH. BMI-for-age (5-19 years) [Available from: https://www.who.int/toolkits/growth-reference-data-for-5to19-years/indicators/bmi-for-age.
38. Organization WH. WHO Regional Offices [Available from: https://www.who.int/about/who-we-are/regional-offices.
39. Cumpston M, Li T, Page MJ, Chandler J, Welch VA, Higgins JP, et al. Updated guidance for trusted systematic reviews: a new edition of the Cochrane Handbook for Systematic Reviews of Interventions. Cochrane Database Syst Rev. 2019;10:ED000142.
40. Van Sluijs EM, Ekelund U, Crochemore-Silva I, Guthold R, Ha A, Lubans D, et al. Physical activity behaviours in adolescence: current evidence and opportunities for intervention. Lancet (in press). 2021.
41. Rahman MT, Nahiduzzaman KM. Examining the Walking Accessibility, Willingness, and Travel Conditions of Residents in Saudi Cities. Int J Environ Res Public Health. 2019;16(4).
42. Goodman A, Paskins J, Mackett R. Day length and weather effects on children's physical activity and participation in play, sports, and active travel. Journal of physical activity & health. 2012;9(8):1105-16.
43. Harrison F, van Sluijs EM, Corder K, Ekelund U, Jones A. The changing relationship between rainfall and children's physical activity in spring and summer: a longitudinal study. Int J Behav Nutr Phys Act. 2015;12:41.
44. Herrador-Colmenero M, Harrison F, Villa-Gonzalez E, Rodriguez-Lopez C, Ortega FB, Ruiz JR, et al. Longitudinal associations between weather, season, and mode of commuting to school among Spanish youths. Scand J Med Sci Sports. 2018;28(12):2677-85.
45. Potoglou D, Arslangulova B. Factors influencing active travel to primary and secondary schools in Wales. Transportation Planning and Technology. 2017;40(1):80-99.
46. Nelson NM, Woods CB. Neighborhood perceptions and active commuting to school among adolescent boys and girls. Journal of physical activity & health. 2010;7(2):257-66.
47. Larouche R, Sarmiento OL, Broyles ST, Denstel KD, Church TS, Barreira TV, et al. Are the correlates of active school transport context-specific? Int J Obes Suppl. 2015;5(Suppl 2):S89-99.
48. Mathews E, Lakshmi JK, Ravindran TK, Pratt M, Thankappan KR. Perceptions of barriers and facilitators in physical activity participation among women in Thiruvananthapuram City, India. Glob Health Promot. 2016;23(4):27-36.
49. Almahmood M, Scharnhorst E, Carstensen TA, Jørgensen G, Schulze O. Mapping the gendered city: investigating the socio-cultural influence on the practice of walking and the meaning of walkscapes among young Saudi adults in Riyadh. Journal of Urban design. 2017;22(2):229-48.
50. Kroesen M, De Vos J. Does active travel make people healthier, or are healthy people more inclined to travel actively? Journal of transport & health. 2020;16:100844.
51. Ostergaard L, Grontved A, Borrestad LA, Froberg K, Gravesen M, Andersen LB. Cycling to school is associated with lower BMI and lower odds of being overweight or obese in a large population-based study of Danish adolescents. Journal of physical activity & health. 2012;9(5):617-25.
52. MacCallum RC, Zhang S, Preacher KJ, Rucker DD. On the practice of dichotomization of quantitative variables. Psychological methods. 2002;7(1):19.

For peer review only - http://bmjopen.bmj.com/site/about/guidelines.xhtml

53. World Development Indicators | DataBank: World Development Indicators. Click on a metadata icon for original source information to be used for citation.; 2021 [Available from: https://databank.worldbank.org/reports.aspx?source=2&series=SE.SEC.UNER.LO.ZS&country=#.
54. Air pollution: Asia's deadliest public health crisis isn't COVID. 2021.
55. UNICEF. Child Friendly Cities and Communities Handbook2018.

For peer review only

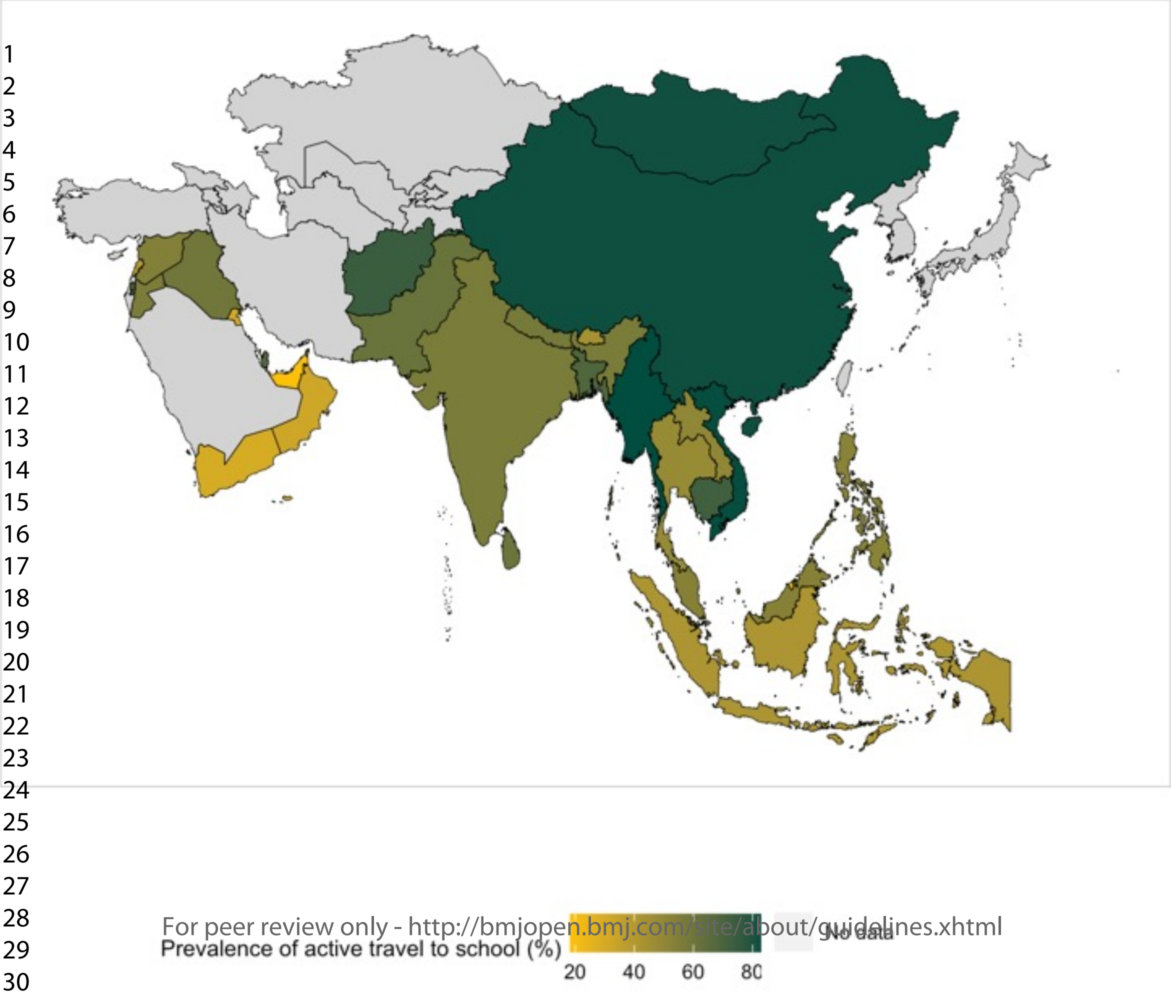

For peer review only - http://bmjopen.bmj.com/site/about/guidelines.xhtml

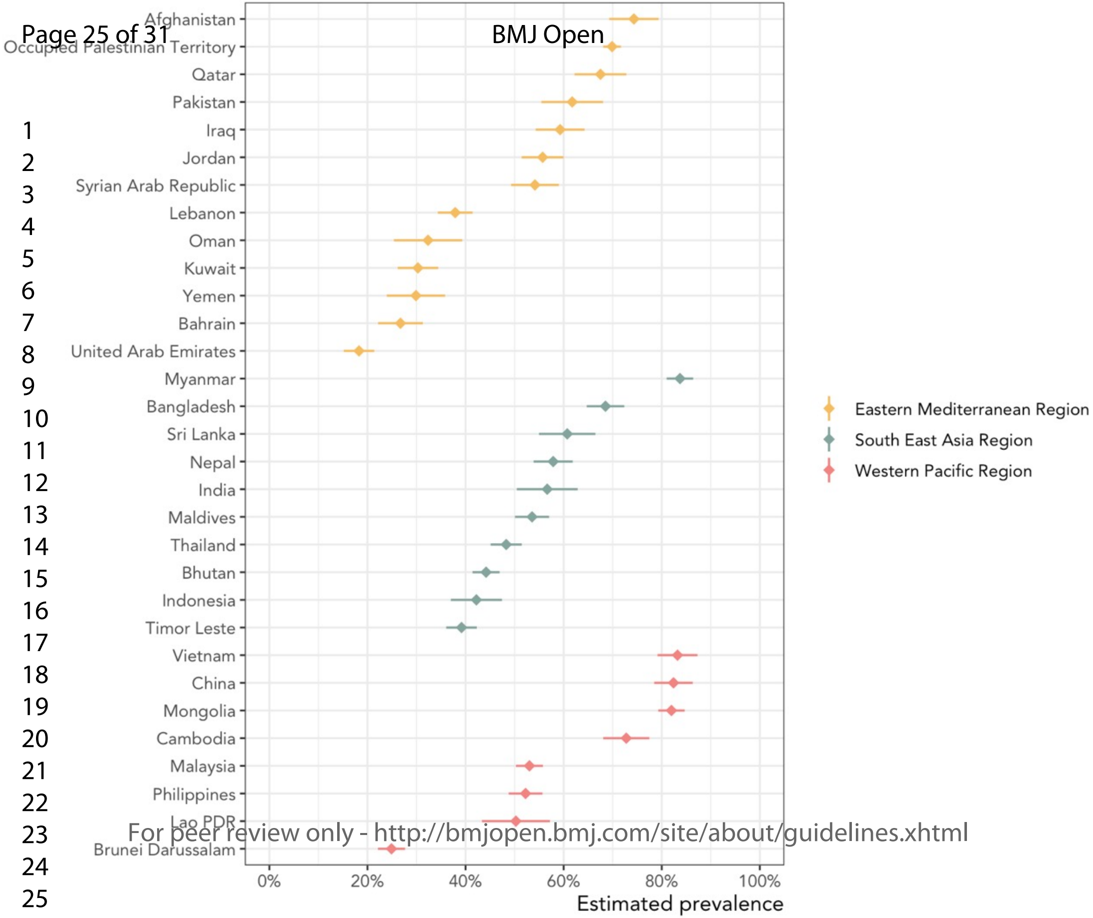

For peer review only - http://bmjopen.bmj.com/site/about/guidelines.xhtml

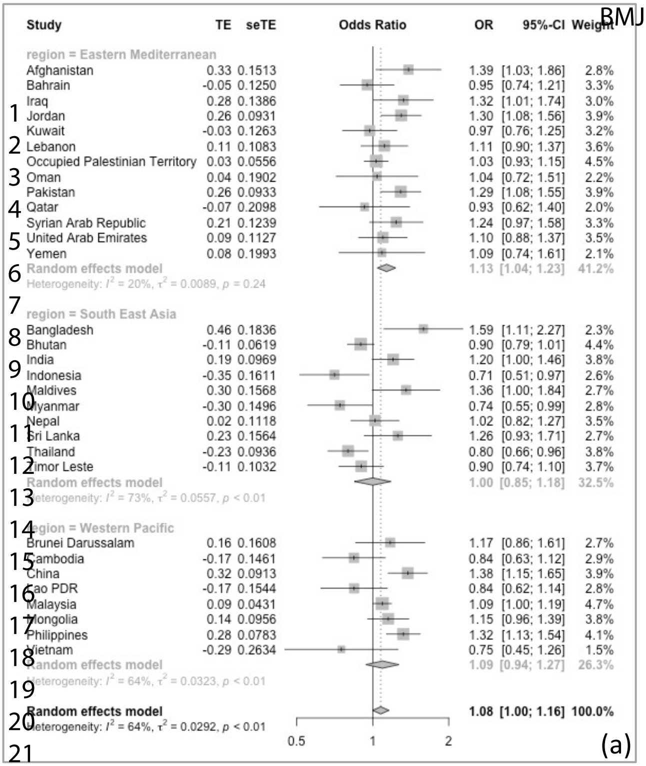
Study TE seTE Odds Ratio OR 95%-CI Weight
region = Eastern Mediterranean
Afghanistan 0.33 0.1513 1.39 [1.03; 1.86] 2.8%
Bahrain -0.05 0.1250 0.95 [0.74; 1.21] 3.3%
Iraq 0.28 0.1386 1.32 [1.01; 1.74] 3.0%
Jordan 0.26 0.0931 1.30 [1.08; 1.56] 3.9%
Kuwait -0.03 0.1263 0.97 [0.76; 1.25] 3.2%
Lebanon 0.11 0.1083 1.11 [0.90; 1.37] 3.6%
Occupied Palestinian Territory 0.03 0.0556 1.03 [0.93; 1.15] 4.5%
Oman 0.04 0.1902 1.04 [0.72; 1.51] 2.2%
Pakistan 0.26 0.0933 1.29 [1.08; 1.55] 3.9%
Qatar -0.07 0.2098 0.93 [0.62; 1.40] 2.0%
Syrian Arab Republic 0.21 0.1239 1.24 [0.97; 1.58] 3.3%
United Arab Emirates 0.09 0.1127 1.10 [0.88; 1.37] 3.5%
Yemen 0.08 0.1993 1.09 [0.74; 1.61] 2.1%
Random effects model 1.13 [1.04; 1.23] 41.2%
Heterogeneity: I² = 20%, τ² = 0.0089, p = 0.24
region = South East Asia
Bangladesh 0.46 0.1836 1.59 [1.11; 2.27] 2.3%
Bhutan -0.11 0.0619 0.90 [0.79; 1.01] 4.4%
India 0.19 0.0969 1.20 [1.00; 1.46] 3.8%
Indonesia -0.35 0.1611 0.71 [0.51; 0.97] 2.6%
Maldives 0.30 0.1568 1.36 [1.00; 1.84] 2.7%
Myanmar -0.30 0.1496 0.74 [0.55; 0.99] 2.8%
Nepal 0.02 0.1118 1.02 [0.82; 1.27] 3.5%
Sri Lanka 0.23 0.1564 1.26 [0.93; 1.71] 2.7%
Thailand -0.23 0.0936 0.80 [0.66; 0.96] 3.8%
Timor Leste -0.11 0.1032 0.90 [0.74; 1.10] 3.7%
Random effects model 1.00 [0.85; 1.18] 32.5%
Heterogeneity: I² = 73%, τ² = 0.0557, p < 0.01
region = Western Pacific
Brunei Darussalam 0.16 0.1608 1.17 [0.86; 1.61] 2.7%
Cambodia -0.17 0.1461 0.84 [0.63; 1.12] 2.9%
China 0.32 0.0913 1.38 [1.15; 1.65] 3.9%
Lao PDR -0.17 0.1544 0.84 [0.62; 1.14] 2.8%
Malaysia 0.09 0.0431 1.09 [1.00; 1.19] 4.7%
Mongolia 0.14 0.0956 1.15 [0.96; 1.39] 3.8%
Philippines 0.28 0.0783 1.32 [1.13; 1.54] 4.1%
Vietnam -0.29 0.2634 0.75 [0.45; 1.26] 1.5%
Random effects model 1.09 [0.94; 1.27] 26.3%
Heterogeneity: I² = 64%, τ² = 0.0323, p < 0.01
Random effects model 1.08 [1.00; 1.16] 100.0%
Heterogeneity: I² = 64%, τ² = 0.0292, p < 0.01
0.5 1 2
(a)

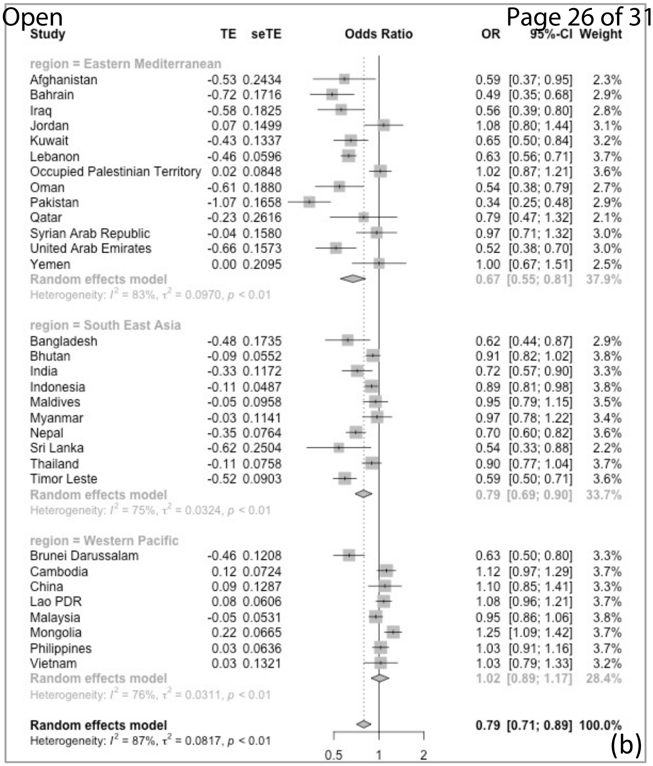
Study TE seTE Odds Ratio OR 95%-CI Weight
region = Eastern Mediterranean
Afghanistan -0.53 0.2434 0.59 [0.37; 0.95] 2.3%
Bahrain -0.72 0.1716 0.49 [0.35; 0.68] 2.9%
Iraq -0.58 0.1825 0.56 [0.39; 0.80] 2.8%
Jordan 0.07 0.1499 1.08 [0.80; 1.44] 3.1%
Kuwait -0.43 0.1337 0.65 [0.50; 0.84] 3.2%
Lebanon -0.46 0.0596 0.63 [0.56; 0.71] 3.7%
Occupied Palestinian Territory 0.02 0.0848 1.02 [0.87; 1.21] 3.6%
Oman -0.61 0.1880 0.54 [0.38; 0.79] 2.7%
Pakistan -1.07 0.1658 0.34 [0.25; 0.48] 2.9%
Qatar -0.23 0.2616 0.79 [0.47; 1.32] 2.1%
Syrian Arab Republic -0.04 0.1580 0.97 [0.71; 1.32] 3.0%
United Arab Emirates -0.66 0.1573 0.52 [0.38; 0.70] 3.0%
Yemen 0.00 0.2095 1.00 [0.67; 1.51] 2.5%
Random effects model 0.67 [0.55; 0.81] 37.9%
Heterogeneity: I² = 83%, τ² = 0.0970, p < 0.01
region = South East Asia
Bangladesh -0.48 0.1735 0.62 [0.44; 0.87] 2.9%
Bhutan -0.09 0.0552 0.91 [0.82; 1.02] 3.8%
India -0.33 0.1172 0.72 [0.57; 0.90] 3.3%
Indonesia -0.11 0.0487 0.89 [0.81; 0.98] 3.8%
Maldives -0.05 0.0958 0.95 [0.79; 1.15] 3.5%
Myanmar -0.03 0.1141 0.97 [0.78; 1.22] 3.4%
Nepal -0.35 0.0764 0.70 [0.60; 0.82] 3.6%
Sri Lanka -0.62 0.2504 0.54 [0.33; 0.88] 2.2%
Thailand -0.11 0.0758 0.90 [0.77; 1.04] 3.7%
Timor Leste -0.52 0.0903 0.59 [0.50; 0.71] 3.6%
Random effects model 0.79 [0.69; 0.90] 33.7%
Heterogeneity: I² = 75%, τ² = 0.0324, p < 0.01
region = Western Pacific
Brunei Darussalam -0.46 0.1208 0.63 [0.50; 0.80] 3.3%
Cambodia 0.12 0.0724 1.12 [0.97; 1.29] 3.7%
China 0.09 0.1287 1.10 [0.85; 1.41] 3.3%
Lao PDR 0.08 0.0606 1.08 [0.96; 1.21] 3.7%
Malaysia -0.05 0.0531 0.95 [0.86; 1.06] 3.8%
Mongolia 0.22 0.0665 1.25 [1.09; 1.42] 3.7%
Philippines 0.03 0.0636 1.03 [0.91; 1.16] 3.7%
Vietnam 0.03 0.1321 1.03 [0.79; 1.33] 3.2%
Random effects model 1.02 [0.89; 1.17] 28.4%
Heterogeneity: I² = 76%, τ² = 0.0311, p < 0.01
Random effects model 0.79 [0.71; 0.89] 100.0%
Heterogeneity: I² = 87%, τ² = 0.0817, p < 0.01
0.5 1 2
(b)

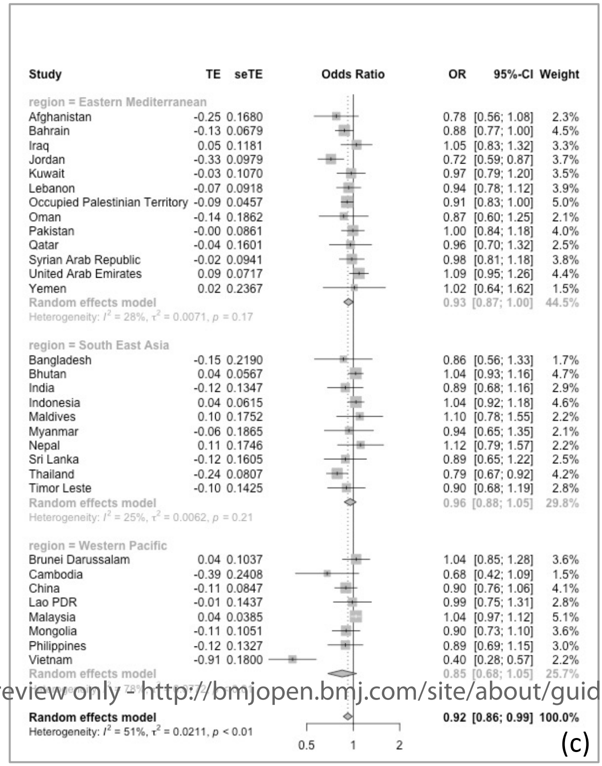
Study TE seTE Odds Ratio OR 95%-CI Weight
region = Eastern Mediterranean
Afghanistan -0.25 0.1680 0.78 [0.56; 1.08] 2.3%
Bahrain -0.13 0.0679 0.88 [0.77; 1.00] 4.5%
Iraq 0.05 0.1181 1.05 [0.83; 1.32] 3.3%
Jordan -0.33 0.0979 0.72 [0.59; 0.87] 3.7%
Kuwait -0.03 0.1070 0.97 [0.79; 1.20] 3.5%
Lebanon -0.07 0.0918 0.94 [0.78; 1.12] 3.9%
Occupied Palestinian Territory -0.09 0.0457 0.91 [0.83; 1.00] 5.0%
Oman -0.14 0.1862 0.87 [0.60; 1.25] 2.1%
Pakistan -0.00 0.0861 1.00 [0.84; 1.18] 4.0%
Qatar -0.04 0.1601 0.96 [0.70; 1.32] 2.5%
Syrian Arab Republic -0.02 0.0941 0.98 [0.81; 1.18] 3.8%
United Arab Emirates 0.09 0.0717 1.09 [0.95; 1.26] 4.4%
Yemen 0.02 0.2367 1.02 [0.64; 1.62] 1.5%
Random effects model 0.93 [0.87; 1.00] 44.5%
Heterogeneity: I² = 28%, τ² = 0.0071, p = 0.17
region = South East Asia
Bangladesh -0.15 0.2190 0.86 [0.56; 1.33] 1.7%
Bhutan 0.04 0.0567 1.04 [0.93; 1.16] 4.7%
India -0.12 0.1347 0.89 [0.68; 1.16] 2.9%
Indonesia 0.04 0.0615 1.04 [0.92; 1.18] 4.6%
Maldives 0.10 0.1752 1.10 [0.78; 1.55] 2.2%
Myanmar -0.06 0.1865 0.94 [0.65; 1.35] 2.1%
Nepal 0.11 0.1746 1.12 [0.79; 1.57] 2.2%
Sri Lanka -0.12 0.1605 0.89 [0.65; 1.22] 2.5%
Thailand -0.24 0.0807 0.79 [0.67; 0.92] 4.2%
Timor Leste -0.10 0.1425 0.90 [0.68; 1.19] 2.8%
Random effects model 0.96 [0.88; 1.05] 29.8%
Heterogeneity: I² = 25%, τ² = 0.0062, p = 0.21
region = Western Pacific
Brunei Darussalam 0.04 0.1037 1.04 [0.85; 1.28] 3.6%
Cambodia -0.39 0.2408 0.68 [0.42; 1.09] 1.5%
China -0.11 0.0847 0.90 [0.76; 1.06] 4.1%
Lao PDR -0.01 0.1437 0.99 [0.75; 1.31] 2.8%
Malaysia 0.04 0.0385 1.04 [0.97; 1.12] 5.1%
Mongolia -0.11 0.1051 0.90 [0.73; 1.10] 3.6%
Philippines -0.12 0.1327 0.89 [0.69; 1.15] 3.0%
Vietnam -0.91 0.1800 0.40 [0.28; 0.57] 2.2%
Random effects model 0.85 [0.68; 1.05] 25.7%
Random effects model 0.92 [0.86; 0.99] 100.0%
Heterogeneity: I² = 51%, τ² = 0.0211, p < 0.01
0.5 1 2
(c)

Supplementary

Supplementary Table S1

Table S1. List of countries, year of surveys, and sample sizes from all 31 Asian countries

Country	Year of survey	Sample size
Eastern Mediterranean		
Afghanistan	2014	2028
Bahrain	2016	6928
Iraq	2012	1878
Jordan	2007	1852
Kuwait	2015	2983
Lebanon	2017	4451
Occupied Palestinian territory	2010	12224
Oman	2015	549
Pakistan	2009	4926
Qatar	2011	763
Syrian Arab Republic	2011	2932
United Arab Emirates	2016	5304
Yemen	2008	804
South East Asia		
Bangladesh	2014	2647
Bhutan	2016	7296
India	2007	6524
Indonesia	2015	10423
Maldives and Male	2009	1668
Myanmar	2016	2534
Nepal	2015	5700
Sri Lanka	2016	1618
Thailand	2015	5440
Timor-Leste	2015	2967
Western Pacific		

For peer review only

Brunei Darussalam	2014	2495
Cambodia	2013	3633
China	2003	7679
Lao People's Democratic Republic	2015	3527
Malaysia	2012	24711
Mongolia	2013	5252
Philippines	2015	7441
Vietnam	2013	3191

For peer review only

Supplementary Figure S1

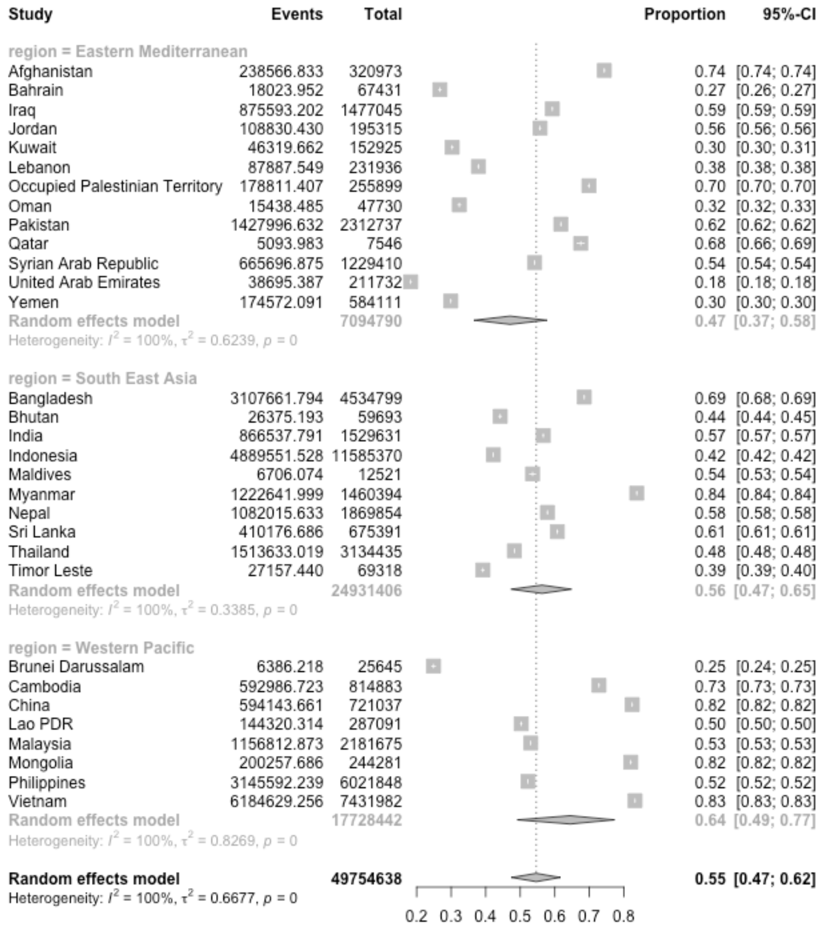

Figure S1. Meta-analysis of prevalence of active travel to school among adolescents in 31 Asian countries

Supplementary Table S2

Meta-regression by year of survey

	**Prevalence**	**Age**	**Sex**	**Weight category**
τ^2 (estimated amount of residual heterogeneity)	0.34	0.023 (SE = 0.01)	0.076 (SE = 0.03)	0.02(SE = 0.01)
τ (square root of estimated tau squared value)	0.58	0.15	0.28	0.13
I^2 (residual heterogeneity / unaccounted variability)	99.99%	61.05%	89.01%	59.97%
H^2 (unaccounted variability / sampling variability)	89240.43	2.57	9.10	2.50
R^2 (amount of heterogeneity accounted for)	N/A	21.77%	7.13%	15.19%
Tests for residual heterogeneity	Wld (df = 19) = 762219.74 (p-value < .0001) LRT (df = 19) = 865578.68 (p-value < .0001)	QE (df = 19) = 43.62 (p-value = 0.01)	QE (df = 19) = 126.65 (p-value < .0001)	QE (df = 19) = 34.83 (p-value = 0.02)
Test of moderators (coefficients 2:12)	QM (df = 11) = 30.03 (p-val = 0.01)	QM (df = 11) = 15.12 (p-val = 0.18)	QM (df = 11) = 12.61 (p-val = 0.32)	QM (df = 11) = 11.36 (p-val = 0.41)

Df = data frame; LRT = likelihood ratio test statistic of the test for (residual) heterogeneity; QE = test statistic for the test of (residual) heterogeneity; QM = test statistic of the omnibus test of moderators; Wld = Wald-type test statistic of the test for residual heterogeneity

For peer review only

STROBE Statement—Checklist of items that should be included in reports of ***cross-sectional studies***

	Item No	Recommendation	Page No
**Title and abstract**	1	(*a*) Indicate the study's design with a commonly used term in the title or the abstract	1
		(*b*) Provide in the abstract an informative and balanced summary of what was done and what was found	2
**Introduction**			
Background/rationale	2	Explain the scientific background and rationale for the investigation being reported	3
Objectives	3	State specific objectives, including any prespecified hypotheses	4
**Methods**			
Study design	4	Present key elements of study design early in the paper	5
Setting	5	Describe the setting, locations, and relevant dates, including periods of recruitment, exposure, follow-up, and data collection	5
Participants	6	(*a*) Give the eligibility criteria, and the sources and methods of selection of participants	5-6
Variables	7	Clearly define all outcomes, exposures, predictors, potential confounders, and effect modifiers. Give diagnostic criteria, if applicable	6
Data sources/ measurement	8*	For each variable of interest, give sources of data and details of methods of assessment (measurement). Describe comparability of assessment methods if there is more than one group	6
Bias	9	Describe any efforts to address potential sources of bias	7
Study size	10	Explain how the study size was arrived at	5
Quantitative variables	11	Explain how quantitative variables were handled in the analyses. If applicable, describe which groupings were chosen and why	6
Statistical methods	12	(*a*) Describe all statistical methods, including those used to control for confounding	6
		(*b*) Describe any methods used to examine subgroups and interactions	7
		(*c*) Explain how missing data were addressed	6
		(*d*) If applicable, describe analytical methods taking account of sampling strategy	7
		(*e*) Describe any sensitivity analyses	7
**Results**			
Participants	13*	(a) Report numbers of individuals at each stage of study—eg numbers potentially eligible, examined for eligibility, confirmed eligible, included in the study, completing follow-up, and analysed	8
		(b) Give reasons for non-participation at each stage	8
		(c) Consider use of a flow diagram	N/A
Descriptive data	14*	(a) Give characteristics of study participants (eg demographic, clinical, social) and information on exposures and potential confounders	8
		(b) Indicate number of participants with missing data for each variable of interest	8
Outcome data	15*	Report numbers of outcome events or summary measures	8
Main results	16	(*a*) Give unadjusted estimates and, if applicable, confounder-adjusted estimates and their precision (eg, 95% confidence interval). Make clear which confounders were adjusted for and why they were included	8-9

For peer review only

		(*b*) Report category boundaries when continuous variables were categorized	8
		(*c*) If relevant, consider translating estimates of relative risk into absolute risk for a meaningful time period	N/A
Other analyses	17	Report other analyses done—eg analyses of subgroups and interactions, and sensitivity analyses	9
**Discussion**			
Key results	18	Summarise key results with reference to study objectives	10
Limitations	19	Discuss limitations of the study, taking into account sources of potential bias or imprecision. Discuss both direction and magnitude of any potential bias	12
Interpretation	20	Give a cautious overall interpretation of results considering objectives, limitations, multiplicity of analyses, results from similar studies, and other relevant evidence	10-12
Generalisability	21	Discuss the generalisability (external validity) of the study results	12
**Other information**			
Funding	22	Give the source of funding and the role of the funders for the present study and, if applicable, for the original study on which the present article is based	14

*Give information separately for exposed and unexposed groups.

Note: An Explanation and Elaboration article discusses each checklist item and gives methodological background and published examples of transparent reporting. The STROBE checklist is best used in conjunction with this article (freely available on the Web sites of PLoS Medicine at http://www.plosmedicine.org/, Annals of Internal Medicine at http://www.annals.org/, and Epidemiology at http://www.epidem.com/). Information on the STROBE Initiative is available at www.strobe-statement.org.

For peer review only

For peer review only - http://bmjopen.bmj.com/site/about/guidelines.xhtml

Descriptive epidemiology of the prevalence of adolescent active travel to school in Asia: a cross-sectional study from 31 countries

Journal:	*BMJ Open*
Manuscript ID	bmjopen-2021-057082.R1
Article Type:	Original research
Date Submitted by the Author:	30-Dec-2021
Complete List of Authors:	Maulida, Rizka; University of Cambridge, MRC Epidemiology Unit; Universitas Indonesia, Faculty of Public Health<br>Ikeda, Erika; University of Cambridge, MRC Epidemiology Unit<br>Oni, T; University of Cambridge, MRC Epidemiology Unit<br>van Sluijs, Esther; University of Cambridge, MRC Epidemiology Unit
**Primary Subject Heading**:	Public health
Secondary Subject Heading:	Epidemiology
Keywords:	PUBLIC HEALTH, EPIDEMIOLOGY, Community child health < PAEDIATRICS

SCHOLARONE™ Manuscripts

I, the Submitting Author has the right to grant and does grant on behalf of all authors of the Work (as defined in the below author licence), an exclusive licence and/or a non-exclusive licence for contributions from authors who are: i) UK Crown employees; ii) where BMJ has agreed a CC-BY licence shall apply, and/or iii) in accordance with the terms applicable for US Federal Government officers or employees acting as part of their official duties; on a worldwide, perpetual, irrevocable, royalty-free basis to BMJ Publishing Group Ltd ("BMJ") its licensees and where the relevant Journal is co-owned by BMJ to the co-owners of the Journal, to publish the Work in this journal and any other BMJ products and to exploit all rights, as set out in our licence.

The Submitting Author accepts and understands that any supply made under these terms is made by BMJ to the Submitting Author unless you are acting as an employee on behalf of your employer or a postgraduate student of an affiliated institution which is paying any applicable article publishing charge ("APC") for Open Access articles. Where the Submitting Author wishes to make the Work available on an Open Access basis (and intends to pay the relevant APC), the terms of reuse of such Open Access shall be governed by a Creative Commons licence – details of these licences and which Creative Commons licence will apply to this Work are set out in our licence referred to above.

Other than as permitted in any relevant BMJ Author's Self Archiving Policies, I confirm this Work has not been accepted for publication elsewhere, is not being considered for publication elsewhere and does not duplicate material already published. I confirm all authors consent to publication of this Work and authorise the granting of this licence.

For peer review only

Descriptive epidemiology of the prevalence of adolescent active travel to school in Asia: a cross-sectional study from 31 countries

Rizka Maulida[1,2], Erika Ikeda[1], Tolu Oni[1], Esther MF van Sluijs[1]

1 MRC Epidemiology Unit, University of Cambridge, Cambridge, UK

2 Department of Epidemiology, Faculty of Public Health, Universitas Indonesia

Correspondence to Rizka Maulida: rizka.maulida@mrc-epid.cam.ac.uk

For peer review only

Abstract

Objective: This study aimed to examine the prevalence of adolescent active travel to school (ATS) across 31 countries and territories in Asia, overall and by age group, sex and Body Mass Index (BMI) category.

Design: Cross-sectional study.

Setting: 31 Asian countries.

Participants: 152,368 13-to-17-year-olds with complete data for age, sex, measured weight and height, and active travel to school from 31 Asian countries from the Global School-based student Health Survey (GSHS).

Primary outcome: Self-reported active travel to school categorized into passive (0 days) and active (1–7 days).

Results: Overall prevalence of adolescent ATS in Asia based on random-effect meta-analysis was 55%, ranging from 18% (United Arab Emirates) to 84% (Myanmar). There was limited sub-regional variation: 47% in the Eastern Mediterranean (EM), 56% in the South East Asia, and 64% in the Western Pacific. Summarized by random-effect meta-analysis, being an older adolescent age 16 years and older (vs. younger age below 16 years: OR=1.08; 95%CI=1.00-1.16) was positively associated with ATS. This association was strongest in EM countries. Summarized by random-effect meta-analysis, females (vs. males: 0.79; 0.71-0.89) and adolescents with overweight/obesity (vs. underweight and normal BMI: 0.92; 0.86-0.99) were less likely to use ATS. Association with sex was strongest in EM countries. Heterogeneity was considerable in all meta-analyses.

Conclusion: The prevalence of adolescent ATS in Asia varies substantially. Overall, older and male adolescents, and adolescents with underweight and normal BMI category are more likely to actively travel to school. However, the main contributor to differences in ATS between and within regions remain unknown. Although there is substantial scope for improving ATS rates in Asia, any policy actions and interventions should be cognisant of local built, social, and natural environmental contexts that may influence active travel behaviour.

For peer review only - http://bmjopen.bmj.com/site/about/guidelines.xhtml

Article Summary

Strengths and limitations of this study

- This study pooled comparable estimates on active travel to school from 152,368 adolescents from 31 countries in Asia
- Data were collected using standardised sampling and data collection methods, enabling comparison across countries and sub-regions
- Data were from low- and middle-income countries; thus, the extent to which these findings are generalisable to other, particularly high-income, countries in Asia is unclear
- Data collection was conducted in schools, and thus the conclusions drawn here are only relevant to adolescents in school

For peer review only

Introduction

Globally, non-communicable diseases (NCDs), notably cancers, cardiovascular diseases, type 2 diabetes and chronic respiratory illness, are responsible for about 40 million deaths each year (1). NCDs have been associated with various modifiable behavioural risk factors including tobacco use, physical inactivity, unhealthy diet and the harmful use of alcohol (1, 2). The risk of developing NCDs can be lowered by avoiding these unhealthy behaviours, for example, by being physically active (3, 4). Physical inactivity-related diseases mostly manifest in adulthood but their development often starts in childhood and adolescence (5).

Studies have reported that health benefits gained from physical activity in adolescence, defined as age 10-19 years (6), are carried forward into adulthood (7-9). Despite these benefits, global research on physical activity using self-reported questionnaires have shown that more than 80% of adolescents are not meeting international guidelines of engaging in 60 minutes of moderate-to-vigorous physical activity on average per day (10). Moreover, evidence suggests that physical activity starts to decline from childhood and continues to decline into adulthood (11-13). By contrast, a continuous high level of physical activity in general, and maintained active travel, for example walking or cycling, throughout adolescence significantly predicts a high level of adult physical activity (14, 15). Therefore, as adolescence is a key period for establishing active living habits, promoting active travel in this period is important to increase overall physical activity among adolescents. Given that the period of adolescence is also the age range for school attendance, active travel to school (ATS) could be an important focus for interventions to increase active travel in this age group.

Previous research in 63 low- and middle-income countries (LMICs) showed that active travel to school is positively associated with adolescents' physical activity (16), supporting the findings of a systematic review (17). A single walk to school has been estimated to contribute between 16-18% of younger adolescents' daily moderate-to-vigorous physical activity (18). However, data on the prevalence of adolescent ATS are scarce. A study using data from the Global Matrix 3.0 showed that half of children and adolescents actively travelled to and from places, where these trips are mainly trips to and/or from school (19). However, this study was unable to generate a pooled estimate of the global prevalence of active travel due to different methodologies used in the countries studied. A more recent study, the 63 LMICs study,

For peer review only - http://bmjopen.bmj.com/site/about/guidelines.xhtml

revealed a prevalence of 56.1%, but the study did not have a specific overall prevalence for Asia as well as comparisons between countries (16). Using a mix of 27 Asian and Pacific countries, another study reported a prevalence of 42.1% (20). Therefore, to date, no studies have solely focused their analyses on Asia, provided data by Asian subregions, nor provided data by BMI (16, 19, 20).

Asia is the largest and most populous continent, with a population of 4.6 billion (21), where many countries are emerging economies. Rapid urbanisation and dynamically changing built environments due to infrastructure development in the region can be obstacles for active travel. Additionally, these obstacles also pose a broader risk to planetary health (22). For example, the cities with the worst air pollution levels globally are within greater Asia (23), and air pollution has negative impacts both on the climate and health, such as respiratory and cardiovascular diseases and cancer (24). Due to the health risks, physical activity outdoors can be unadvisable. Therefore, future interventions for active travel in this region will have to consider the specific characteristics of built environment and air pollution trends. However, interventions need to be based on an understanding of the pre-existing prevalence in this population group and region; the paucity of evidence on the prevalence of ATS across Asia poses a challenge for evidence-informed policies to promote ATS.

A few studies have been conducted in limited East and Southeast Asian countries. However, these studies had relatively small sample sizes (between 330 and 1518 participants) (25-30), were not able to compare across regions or look at differences by age or Body Mass Index (BMI) category. Evidence from these limited Asian studies suggests that boys are more likely to use ATS compared to girls, and students living in less wealthy areas or studying in less wealthy areas are more likely to use active travel than their wealthier counterparts. ATS is also positively associated with (perceived and objectively measured) shorter distance to school, favourable built environments (e.g., larger street block size and tree cover), and higher social interaction (26-30).

The goal of this study was to examine the prevalence of adolescent ATS across 31 countries and territories in Asia, overall and by age group, sex and BMI category.

For peer review only - http://bmjopen.bmj.com/site/about/guidelines.xhtml

Methods

The reporting of this paper follows the Strengthening the Reporting of OBservational studies in Epidemiology (STROBE) guideline (31).

Data source

This study used data from the Global School-based Student Health Survey (GSHS), developed by the World Health Organization (WHO) and the Center for Disease Control and Prevention (CDC) in collaboration with UNICEF, UNESCO, and UNAIDS (32). The GSHS is a school-based survey conducted mostly among students aged 13–17 years, but some countries also include those aged 11–12 and 18 years (33). Detailed information about the GSHS can be found on WHO and CDC websites (34, 35).

GSHS used a two-staged cluster sampling design to obtain a nationally representative sample of the adolescents. In the first stage, schools were randomly selected using the probability proportional to size sampling. In the second stage, classes were randomly selected in the selected schools, with varied number of classes depending on the school-size. All students in the selected classes were eligible to participate in the survey, and the participation was anonymous and voluntary.

The GSHS surveys have been conducted in 185 mostly low- and middle-income countries globally from 2003 to 2017. The survey consists of 10 core modules on adolescent health behaviours including physical activity and nutrition and protective factors, along with other optional modules. Data on response rates from each survey and characteristics of non-respondents were not available. Data cleaning and management, or data edits, were performed on all GSHS datasets: out of range edits, multiple response edits, logical consistency edits, height and weight edits, variable edits, and record-level edits, where when the responses did not meet the requirement, they were set to missing. Observations with missing data were kept in the datasets. All GSHS datasets are freely available on the WHO's website: https://www.who.int/teams/noncommunicable-diseases/surveillance/systems-tools/global-school-based-student-health-survey/.

Study design and ethics

This cross-sectional study used GSHS data from 31 Asian countries, as defined by the United Nations' Statistics Division of geographic regions (36). All 31 countries assessed ATS

For peer review only - http://bmjopen.bmj.com/site/about/guidelines.xhtml

using GSHS surveys. The GSHS survey in each country was approved by the Ministry of Education, Ministry of Health, or other institution in charge of the survey and underwent ethical clearance. Only those adolescents and their parents who provided written or verbal consent participated. As the current study used retrospective, de-identified, publicly available data, ethics approval was not required for this secondary analysis.

Data management

Data management was done using RStudio version 1.4. Prior to WHO data publication, out of range edits, multiple response edits, logical consistency edits, height and weight edits, variable edits, and record-level edits were performed on all GSHS datasets (33). All 31 country datasets were drawn from the most recent GSHS survey in each country, except for Maldives and Oman which did not have national datasets for physical activity components in their most recent datasets. All 31 datasets were checked for missing values and observations with missing values were dropped.

Study variables

The outcome variable, active travel to school, was self-reported using the question "During the past seven days, on how many days did you walk or ride a bicycle to or from school?" with eight standard GSHS responses ranging from 0-7 days. In this study, travel to school was dichotomised into *passive* (0 days) and *active* (1-7 days) travel. This categorisation was chosen based on the distribution of ATS across the dataset (Supplementary Figure S1) which showed high counts of 0 days, small counts of 1-4 days, and higher counts of 5-7 days.

Independent variables included age, sex, and BMI category. Age (in years) and sex (male or female) were self-reported. For analysis, age was categorised into two groups: *younger* (under 16 years old) and *older* (16 years old and over) adolescents. This cut-off point was as per standard GSHS reporting (37). Sex was expressed as ‘1’ for *male*, and ‘2’ for *female*. Participants’ height and weight were measured by survey staff before survey administration. Measurements were written by staff on slips of paper and given to each participant to be entered into their GSHS answer sheet. BMI category was generated based on the WHO categorisation of Body Mass Index (BMI)-for-age among 5–19-year-old-children, and then categorised into: *underweight and normal BMI* or *overweight and obesity* (38).

For peer review only - http://bmjopen.bmj.com/site/about/guidelines.xhtml

Statistical analysis

All statistical analyses were conducted using RStudio version 1.4. Only participants with complete data were included in the analyses (ranging from 34.2% to 97.4% by country). The 31 countries were categorised into three sub-regions: Eastern Mediterranean (which mostly consists of Middle Eastern countries); South East Asia (which consists of countries in the South and some countries in the Southeast Asia region); and Western Pacific (which consists of the rest of the countries in the Southeast Asia region along with countries in East and Central Asia). This categorisation was based on cultural and climate similarities shared by these countries, as well as WHO regional offices (39). To adjust for nonresponse and distribution of adolescents by cluster, a sampling (survey) weight was applied to each adolescent. These weights were generated by accounting the size of cluster where the adolescent was sampled. Percentages and association estimates were also calculated under these weights to be representative of all students in each cluster. Logistic regression modelling was used to estimate each country's weighted associations of ATS, as independent variable, with age group, sex and BMI category, as dependent variables. Younger (<16 years old) adolescents, male adolescents, and adolescents with underweight or normal BMI category were used as the reference category. The weighted percentages and estimates (i.e., odds ratio (OR)) were pooled using random-effects meta-analysis to obtain the overall prevalence and estimates for the 31 countries. I^2 was used to determine the importance of heterogeneity in each meta-analysis (0-40%: might not be important, 30-60%: may represent moderate heterogeneity, 50-90%: may represent substantial heterogeneity, 75-100%: considerable heterogeneity) (40). We stratified the meta-analysis by sub-region to establish potential regional differences. We also performed meta-regression to check the potential impact of year of survey variability. Year of survey, and sample size are shown on Supplementary Table S1.

Patient and public involvement

Patients or the public WERE NOT involved in the design, or conduct, or reporting, or dissemination plans of our research.

For peer review only - http://bmjopen.bmj.com/site/about/guidelines.xhtml

Results

Of the original 174,449 adolescents surveyed from the 31 countries, there were 152,368 (87.3%) adolescents aged 11-18 years with complete data included in the analysis. Participants were excluded due to missing values in one or more variables: n=893 for age, n=1,518 for sex, n=19,996 for weight and height, and n=2,866 for travel to school. There was no significant difference between those included in this analysis, those excluded from this study, and those excluded based on weight and height variables (Supplementary Table S2). Table 1 shows the characteristics of adolescents included in the analyses, stratified by country. The proportion of younger adolescents varied across countries: from 50% in Thailand to 15% in Lao People's Democratic Republic. Across all countries, percentages of male participants were relatively equal, ranging between 45% and 50%. With respect to BMI category, Kuwait had the highest percentage of adolescents with overweight and obesity (51%), and Vietnam (6.2%) and Timor Leste (6.2%) had the lowest.

Overall active travel to school prevalence

The overall prevalence of adolescent ATS in Asia was 55.0% (see forest plot in Supplementary Figure S2). Figure 1 and Figure 2 show a map and a forest plot of the weighted prevalence of ATS in the 31 Asian countries, respectively. The forest plot is stratified by sub-region and sorted from highest to lowest prevalence. In the Eastern Mediterranean, the ATS prevalence ranged from 18% (United Arab Emirates) to 74.0% (Afghanistan), with a median value of 54%. In the South East Asia, the prevalence of ATS ranged from 39% (Timor Leste) to 84% (Myanmar), with a median value of 56%. In the Western Pacific, ATS prevalence ranged from 25.0% (Brunei Darussalam) to 82.0% (China), with a median value of 63.0%.

Active travel to school association with age, sex, and BMI category

Overall, older adolescents were 8% more likely to actively travel to school compared to younger adolescents (OR: 1.08; 95% CI: 1.00, 1.16) (Figure 3a). This association was strongest in the Eastern Mediterranean countries (OR: 1.13; 95% CI: 1.04, 1.23), whereas no association was observed in the other two Asian sub-regions. Figure 3b shows that female adolescents were 21% less likely to actively travel to school than male adolescents (OR: 0.79; 95% CI: 0.71, 0.89). Stratified analyses showed that this association was strongest in Eastern

For peer review only - http://bmjopen.bmj.com/site/about/guidelines.xhtml

Mediterranean countries (OR: 0.67; 95% CI: 0.55, 0.81), and then in South-East Asia countries (OR: 0.79; 95% CI: 0.69, 0.90). No association was observed in Western Pacific countries. Lastly, adolescents with overweight and obesity were 8% less likely to actively travel to school than those with underweight or normal (OR: 0.92; 95% CI: 0.86, 0.99) (Figure 3c) weight categories. However, stratified analyses did not show any statistically significant associations by sub-region.

Heterogeneity

Overall heterogeneity (I^2) was considerable in all three meta-analyses (age group: 64%, sex: 87%, and BMI category: 51%) (Figure 3). Some region-specific variations were observed, with only the association with sex showing considerable heterogeneity (83%) in the Eastern Mediterranean; whereas considerable heterogeneity was observed across all meta-analyses in the Western Pacific. Meta-regression shows that year of survey accounted for some of this heterogeneity (Supplementary Table S3).

For peer review only

Discussion

Main findings

This study showed that 55% of Asian adolescents walk or cycle to school at least once per week. This prevalence varied substantially by country, with estimates varying from 18% (United Arab Emirates) to 84% (Myanmar). However, regional variation was limited with 54% ATS prevalence noted in the Eastern Mediterranean, 55.5% in the South East Asia, and 63% in the Western Pacific. Overall, older adolescents, male adolescents, and adolescents with underweight/normal weight were more likely to use active modes than their counter parts. However, these associations varied across countries, suggesting that there may be substantial country-level variation in determinants of ATS.

Prevalence of active travel to school

The prevalence observed in the current study (55%) is broadly similar to that reported in previous studies from various countries and regions (16, 19, 20). However, this is lower than global estimates in a recent review, which reported that 62% of boys and 54% girls actively travel to school (41). Due to methodological differences between studies, our study's prevalence can only be compared directly to prevalence obtained from other GSHS studies (16, 20) and comparison to other studies should be done with caution.

Despite limited regional variation, the Eastern Mediterranean had the lowest prevalence of adolescent ATS, which might reflect the high prevalence of physical inactivity in the general adolescent population in Eastern Mediterranean countries (10). High physical inactivity, which includes ATS, may be due to the climate in the region. Evidence from research on adults in Saudi Arabia showed that half of participants were hesitant to walk daily due to the hot weather during the summer (42). However, this evidence did not show that the hesitancy changed with changing seasons. Furthermore, other studies of children and adolescents conducted in European countries have shown that temperature, precipitation, and wind speed were associated with various domains of physical activity, including ATS (43-45).

For peer review only - http://bmjopen.bmj.com/site/about/guidelines.xhtml

For peer review only

Differences by age, sex and BMI category

Similar to our findings, a study in Hong Kong reported that older adolescents were more likely to actively travel to school (30). However, studies in other Asian countries have not reported differences in active travel between age groups (26-29). Coincidentally, most of these previous studies come from Asian countries belonging to the Western Pacific, where we also found no difference of ATS prevalence between younger and older adolescents. In our study, difference in ATS prevalence by age group was also not noted in South East Asia countries but only present in Eastern Mediterranean countries. A plausible explanation could be that the age when adolescents are permitted to travel independently differ by countries, perhaps due to varying perceptions of risks of injury or violence. For instance, age for independent mobility in South East Asia and Western Pacific countries may be lower than our study's age categorisation cut-off point. However, to our knowledge, no studies have explored this for the Asian region.

Findings from both our study and previous research using the GSHS and other global datasets demonstrated that female adolescents in Asia are less likely to actively travel to school than male adolescents (25, 27, 46, 47). Similarly, among United States of America and European adolescents, female adolescents accumulated lower overall physical activity compare to male adolescents (41). In general, female adolescents in Asia are more restricted in active travel due to cultural norms (48). For example, a study among Indian adults showed that cycling is deemed appropriate for men but improper and unacceptable for women (49). Another study from Saudi Arabia also reported that women's presence on the streets is limited because urban streets are considered a domain for men (50). These cultural expectations may act as a barrier to increasing the prevalence of ATS amongst adolescent females in Asia.

Although adolescents with overweight and obesity BMI category were less likely to actively travel to school, this association was not observed in the sub-region stratified analyses. Strong associations were only present in Cambodia and Vietnam, which is similar to findings from a Dutch population study where BMI was found to negatively influence later levels of active travel (51). The results of our study suggest that BMI category may not a significant determinant of ATS in most Asian countries. A plausible explanation for this finding could be that those adolescents with overweight and obesity BMI category are equally motivated to engage in active travel to obtain healthier BMI. However, the association of BMI

For peer review only - http://bmjopen.bmj.com/site/about/guidelines.xhtml

and active travel may also be bi-directional. For example, a Danish adolescent study reported that cycling to school was associated with lower BMI (52).

Strengths and limitations

This study pooled estimates from 152,368 adolescents from 31 countries in Asia. Sub-region and age-, sex- and BMI-specific estimates were also analysed. Data were collected using standardised sampling and data collection methods, enabling comparison across countries and sub-regions. However, the prevalence of ATS was obtained from one single self-reported question on both walking and cycling to school. Participating countries in the GSHS were also mostly those categorised as low- and middle-income countries in Asia. As a result, the extent to which these findings are generalisable to other, particularly high-income, countries in Asia is unclear. This study also dichotomised both dependent and independent variables for meta-analysis purposes, which may have contributed to biases in the results, such as loss of information about individual differences and loss of effect (53). Data on response rates from each survey and characteristics of non-respondents were not available, but we used the study weights provided to obtain representative estimates. Data collection was conducted in schools, and thus the conclusions drawn here are only relevant to adolescents in school, however out of school adolescents account for approximately 7% in Asia as compared to global percentage of 15% (54). The GSHS also mainly focuses on adolescents aged 13-17 years old, and the proportion of 11-12 years old were much smaller. Therefore, the evidence from adolescents in this younger age group may be underrepresented. Lastly, the GSHS data set does not contain information on individual-level socio-economic position, which may be an important determinant of ATS.

Implications

This evidence shows that there is substantial scope for improving ATS prevalence in Asian countries, which less than half of adolescents using ATS in many countries. The prevalence of ATS varied between and within the three Asian sub-regions, as did the associations with individual factors (i.e., age group, sex and BMI category). This suggests that targeted promotion to certain population subgroups may be useful. However, future research is needed to explore why these variations occur among these countries to inform future policies and practices on ATS. Noting the variation of ATS by age group among the countries,

For peer review only - http://bmjopen.bmj.com/site/about/guidelines.xhtml

age of independent mobility, especially on Eastern Mediterranean countries, should be studied so that future interventions with focus of age groups are appropriately designed. Future interventions should also focus more on designing ATS interventions that target female adolescents in Eastern Mediterranean and South East Asia countries. Any policy actions or interventions will need to be contextually sensitive, cognisant of local built, social, and natural environmental contexts that could influence ATS. Further studies are therefore needed to gather evidence on the roles that environmental factors, such as exposure to air pollution, high density traffic, walkability, and safety, play in influencing ATS across Asian countries (55). Such studies could build on child-friendly city initiatives (56) to generate evidence to inform strategies to equitably improve adolescent ATS across the region. Furthermore, there is a need to explore gendered cultural norms in the context of Asian countries, to ensure interventions designed to encourage ATS do not exclude female adolescents.

Conclusions

The overall prevalence of adolescent ATS in Asia was 55%. The prevalence was lowest in United Arab Emirates and highest in Myanmar. Overall, older adolescents, male adolescents, and adolescents with underweight and normal BMI were more likely to actively travel to school than their counterparts. Although age, sex, and BMI status were associated with the prevalence of ATS to varying degrees in the Eastern Mediterranean, South East Asia, and Western Pacific regions, the main driver of variation within and between Asian countries remains unknown. Further investigations to identify other potential factors which account for differences in adolescent ATS prevalence across Asian countries is therefore needed to inform policy and practice.

For peer review only

Declarations

Funding

RM's doctoral study is supported by Indonesia Endowment Fund for Education (201908220815174). EMFvS and EI are supported by the Medical Research Council [grant number MC_UU_00006/5]. TO is supported by the National Institute for Health Research (NIHR) (16/137/34) using UK aid from the UK Government to support global health research.

Contributors

RM designed the study with the supervision of EMFvS and TO. RM collected and managed the data assisted by EI. RM conducted the statistical analysis with guidance from EMFvS and EI. RM drafted the paper. All authors reviewed the results, edited the manuscript and agreed on the final version of the manuscript.

Ethic statement

In each individual country, the GSHS was approved by the Ministry of Education, Ministry of Health, or other institution in charge of the survey. Ethical approval was also obtained in all 31 individual countries. No reference numbers for these ethical approvals are available. Only those adolescents and their parents who provided written or verbal consent participated. As the current study used retrospective, de-identified, publicly available data, no separate ethics approval was required for the analysis of the data.

Competing interests

None declared.

Data sharing statement

Original GSHS datasets are available publicly online at https://www.who.int/teams/noncommunicable-diseases/surveillance/systems-tools/global-school-based-student-health-survey. Processed datasets used for this study and R codes used for data management and data analysis are available by emailing Rizka Maulida at rizka.maulida@mrc-epid.cam.ac.uk

For peer review only

Disclaimer

The views expressed in this publication are those of the authors and not necessarily those of the funders. The funders had no role in the study.

Map disclaimer

The depiction of boundaries on the map(s) in this article does not imply the expression of any opinion whatsoever on the part of BMJ (or any member of its group) concerning the legal status of any country, territory, jurisdiction or area or of its authorities. The map(s) are provided without any warranty of any kind, either express or implied.

Acknowledgements

The authors would like to thank Stephen Sharp of MRC Epidemiology Unit, University of Cambridge and Ihsan Fadilah of Eijkman-Oxford Clinical Research Unit, Jakarta, for the support on data analysis, and Bimandra Djaafara of Imperial College, London, for the assistance on map creation.

Twitter

Rizka Maulida @rizkamaulida13, Erika Ikeda @DrErikaIkeda, Tolu Oni @DrTolullah and Esther MF van Sluijs @EvanSluijs

For peer review only

Table 1. Characteristic of adolescents included in analyses from all 31 Asian countries

Country	Population[a]	Age category[b]		Sex		BMI category[c]	
		Younger	Older	Male	Female	Underweight and normal	Overweight and obesity
Eastern Mediterranean							
Afghanistan	320,973	34%	66%	54%	46%	83%	17%
Bahrain	67,431	54%	46%	50%	50%	58%	42%
Iraq	1,477,045	51%	49%	57%	43%	73%	27%
Jordan	195,315	37%	63%	52%	48%	76%	24%
Kuwait	152,925	36%	64%	51%	49%	49%	51%
Lebanon	231,936	44%	56%	46%	54%	72%	28%
Occupied Palestinian territory	255,899	70%	30%	49%	51%	74%	26%
Oman	47,730	30%	70%	47%	53%	76%	24%
Pakistan	2,312,737	61%	39%	61%	39%	92%	8.1%
Qatar	7,546	81%	19%	52%	48%	50%	50%
Syrian Arab Republic	1,229,410	75%	25%	51%	49%	73%	27%
United Arab Emirates	211,732	38%	62%	49%	51%	59%	41%
Yemen	584,111	57%	43%	64%	36%	88%	12%
South East Asia							
Bangladesh	4,534,799	64%	36%	64%	36%	89%	11%

For peer review only - http://bmjopen.bmj.com/site/about/guidelines.xhtml

Bhutan	59,693	29%	71%	48%	52%	87%	13%
India	1,529,631	65%	35%	58%	42%	88%	12%
Indonesia	11,585,370	67%	33%	48%	52%	82%	18%
Maldives and Male	12,521	35%	65%	49%	51%	88%	12%
Myanmar	1,460,394	65%	35%	45%	55%	92%	8.4%
Nepal	1,869,854	54%	46%	48%	52%	92%	8.0%
Sri Lanka	675,391	39%	61%	46%	54%	85%	15%
Thailand	3,134,435	50%	50%	45%	55%	81%	19%
Timor-Leste	69,318	22%	78%	50%	50%	94%	6.2%
Western Pacific							
Brunei Darussalam	25,645	49%	51%	49%	51%	63%	37%
Cambodia	814,883	31%	69%	52%	48%	96%	3.8%
China	721,037	73%	27%	51%	49%	82%	18%
Lao People's Democratic Republic	287,091	15%	85%	53%	47%	88%	12%
Malaysia	2,181,675	41%	59%	50%	50%	76%	24%
Mongolia	244,281	49%	51%	48%	52%	88%	12%
Philippines	6,021,848	49%	51%	49%	51%	90%	10%
Vietnam	7,431,982	21%	79%	47%	53%	94%	6.2%

[a] population represented by the sample; [b] age was categorised into younger (< 16 years old), older (≥ 16); [c] BMI category was dichotomised by WHO categorisation of BMI-for-age among 5-19-year-old children.

For peer review only - http://bmjopen.bmj.com/site/about/guidelines.xhtml

Figure 1. Weighted prevalence of active travel to school in adolescents from 31 Asian countries using data from Global School-based Student Health Survey 2003 to 2017

Figure 2. Weighted prevalence of active travel to school across 31 Asian countries using data from Global School-based Student Health Survey 2003 to 2017

Figure 3. Meta-analyses of logistic regressions for the associations of active travel to school and age, with younger adolescent as reference group (a), sex, with male as the reference group (b), and BMI category, with underweight and normal BMI as the reference group (c) in 31 Asian countries using data from Global School-based Student Health Survey 2003 to 2017

For peer review only

References

1. WHO. Non communicable diseases 2018 [Available from: http://www.who.int/news-room/fact-sheets/detail/noncommunicable-diseases.
2. Collaborators GBDRF. Global, regional, and national comparative risk assessment of 79 behavioural, environmental and occupational, and metabolic risks or clusters of risks, 1990-2015: a systematic analysis for the Global Burden of Disease Study 2015. Lancet. 2016;388(10053):1659-724.
3. Beaglehole R, Yach D. Globalisation and the prevention and control of non-communicable disease: the neglected chronic diseases of adults. Lancet. 2003;362(9387):903-8.
4. Collaborators GBDRF. Global, regional, and national comparative risk assessment of 84 behavioural, environmental and occupational, and metabolic risks or clusters of risks, 1990-2016: a systematic analysis for the Global Burden of Disease Study 2016. Lancet. 2017;390(10100):1345-422.
5. Parsons TJ, Power C, Logan S, Summerbell CD. Childhood predictors of adult obesity: a systematic review. Int J Obes Relat Metab Disord. 1999;23 Suppl 8:S1-107.
6. Organization WH. Adolescence: a period needing special attention - recognizing-adolescence. 2021.
7. Sallis JF, Owen N. Physical Activity and Behavioral Medicine: SAGE Publications; 1999.
8. WHO. Global Recommendations on Physical Activity for Health. Global Recommendations on Physical Activity for Health. WHO Guidelines Approved by the Guidelines Review Committee. Geneva2010.
9. Dick B, Ferguson BJ. Health for the world's adolescents: a second chance in the second decade. J Adolesc Health. 2015;56(1):3-6.
10. Guthold R, Stevens GA, Riley LM, Bull FC. Global trends in insufficient physical activity among adolescents: a pooled analysis of 298 population-based surveys with 1.6 million participants. The Lancet Child & adolescent health. 2019.
11. Farooq MA, Parkinson KN, Adamson AJ, Pearce MS, Reilly JK, Hughes AR, et al. Timing of the decline in physical activity in childhood and adolescence: Gateshead Millennium Cohort Study. British journal of sports medicine. 2018;52(15):1002-6.
12. Corder K, Winpenny E, Love R, Brown HE, White M, Sluijs EV. Change in physical activity from adolescence to early adulthood: a systematic review and meta-analysis of longitudinal cohort studies. British journal of sports medicine. 2019;53(8):496-503.
13. Winpenny EM, Smith M, Penney T, Foubister C, Guagliano JM, Love R, et al. Changes in physical activity, diet, and body weight across the education and employment transitions of early adulthood: A systematic review and meta-analysis. Obes Rev. 2020;21(4):e12962.
14. Telama R, Yang X, Viikari J, Valimaki I, Wanne O, Raitakari O. Physical activity from childhood to adulthood: a 21-year tracking study. Am J Prev Med. 2005;28(3):267-73.
15. Yang X, Telama R, Hirvensalo M, Tammelin T, Viikari JS, Raitakari OT. Active commuting from youth to adulthood and as a predictor of physical activity in early midlife: the young Finns study. Preventive Medicine. 2014;59:5-11.
16. Peralta M, Henriques-Neto D, Bordado J, Loureiro N, Diz S, Marques A. Active Commuting to School and Physical Activity Levels among 11 to 16 Year-Old Adolescents from 63 Low- and Middle-Income Countries. Int J Environ Res Public Health. 2020;17(4).

For peer review only - http://bmjopen.bmj.com/site/about/guidelines.xhtml

17. Larouche R, Saunders TJ, Faulkner G, Colley R, Tremblay M. Associations between active school transport and physical activity, body composition, and cardiovascular fitness: a systematic review of 68 studies. Journal of physical activity & health. 2014;11(1):206-27.
18. Southward EF, Page AS, Wheeler BW, Cooper AR. Contribution of the school journey to daily physical activity in children aged 11-12 years. Am J Prev Med. 2012;43(2):201-4.
19. Gonzalez SA, Aubert S, Barnes JD, Larouche R, Tremblay MS. Profiles of Active Transportation among Children and Adolescents in the Global Matrix 3.0 Initiative: A 49-Country Comparison. Int J Environ Res Public Health. 2020;17(16).
20. Uddin R, Mandic S, Khan A. Active commuting to and from school among 106,605 adolescents in 27 Asia-Pacific countries. Journal of transport & health. 2019;15:100637.
21. United Nations DoEaSA, Population Division. World Population Prospects 2019 2019 [Available from: https://population.un.org/wpp/.
22. Prior JH, Connon IL, McIntyre E, Adams J, Capon A, Kent J, et al. Built environment interventions for human and planetary health: integrating health in climate change adaptation and mitigation. Public Health Res Pract. 2018;28(4).
23. IQAir. 2020 World Air Quality Report. 2021.
24. Manisalidis I, Stavropoulou E, Stavropoulos A, Bezirtzoglou E. Environmental and Health Impacts of Air Pollution: A Review. Frontiers in public health. 2020;8:14.
25. Tudor-Locke C, Ainsworth BE, Adair LS, Popkin BM. Objective physical activity of filipino youth stratified for commuting mode to school. Med Sci Sports Exerc. 2003;35(3):465-71.
26. Lin J-J, Chang H-T. Built Environment Effects on Children's School Travel in Taipai: Independence and Travel Mode. Urban Studies. 2009;47(4):867-89.
27. Trang NH, Hong TK, Dibley MJ. Active commuting to school among adolescents in Ho Chi Minh City, Vietnam: change and predictors in a longitudinal study, 2004 to 2009. Am J Prev Med. 2012;42(2):120-8.
28. Leung A, Le TPL. Factors associated with adolescent active travel: A perceptive and mobility culture approach – Insights from Ho Chi Minh City, Vietnam. Transportation Research Part A: Policy and Practice. 2019;123:54-67.
29. Barnett A, Akram M, Sit CH, Mellecker R, Carver A, Cerin E. Predictors of healthier and more sustainable school travel mode profiles among Hong Kong adolescents. Int J Behav Nutr Phys Act. 2019;16(1):48.
30. Leung KYK, Loo BPY. Determinants of children's active travel to school: A case study in Hong Kong. Travel Behaviour and Society. 2020;21:79-89.
31. STROBE Statement—Checklist of items that should be included in reports of cross-sectional studies 2009 [Available from: https://www.strobe-statement.org/fileadmin/Strobe/uploads/checklists/STROBE_checklist_v4_cross-sectional.pdf.
[dataset] 32. World Health Organization. Data from: Global School-based Student Health Survey. WHO NCD Microdata Repository. 2019.
33. US Centers for Disease Control and Prevention, World Health Organization. GSHS data user's guide. 2015.
34. Global School-based Student Health Survey (GSHS) 2019 [updated 2019-05-23T02:57:19Z/. Available from: https://www.cdc.gov/gshs/index.htm.
35. Larsen K, Buliung R, Faulkner G. Safety and school travel: How does the environment along the route relate to safety and mode choice? Transp Res Rec [Internet]. 2013 Jan 1

For peer review only - http://bmjopen.bmj.com/site/about/guidelines.xhtml

[cited 2016 Nov 1]; 2327(1):[9-18 pp.]. Available from: https://journals.sagepub.com/doi/10.3141/2327-02.
36. Division UNS. Methodology: Standard country or area codes for statistical use (M49) 2020 [Available from: https://unstats.un.org/unsd/methodology/m49/.
37. Regions - CDC Global School-based Student Health Survey (GSHS) 2019 [updated 2019-08-09T01:12:12Z/. Available from: https://www.cdc.gov/gshs/countries/index.htm.
38. World Health Organization. BMI-for-age (5-19 years) [Available from: https://www.who.int/toolkits/growth-reference-data-for-5to19-years/indicators/bmi-for-age.
39. World Health Organization. WHO Regional Offices [Available from: https://www.who.int/about/who-we-are/regional-offices.
40. Cumpston M, Li T, Page MJ, Chandler J, Welch VA, Higgins JP, et al. Updated guidance for trusted systematic reviews: a new edition of the Cochrane Handbook for Systematic Reviews of Interventions. Cochrane Database Syst Rev. 2019;10:ED000142.
41. Van Sluijs EM, Ekelund U, Crochemore-Silva I, Guthold R, Ha A, Lubans D, et al. Physical activity behaviours in adolescence: current evidence and opportunities for intervention. Lancet (in press). 2021.
42. Rahman MT, Nahiduzzaman KM. Examining the Walking Accessibility, Willingness, and Travel Conditions of Residents in Saudi Cities. Int J Environ Res Public Health. 2019;16(4).
43. Goodman A, Paskins J, Mackett R. Day length and weather effects on children's physical activity and participation in play, sports, and active travel. Journal of physical activity & health. 2012;9(8):1105-16.
44. Harrison F, van Sluijs EM, Corder K, Ekelund U, Jones A. The changing relationship between rainfall and children's physical activity in spring and summer: a longitudinal study. Int J Behav Nutr Phys Act. 2015;12:41.
45. Herrador-Colmenero M, Harrison F, Villa-Gonzalez E, Rodriguez-Lopez C, Ortega FB, Ruiz JR, et al. Longitudinal associations between weather, season, and mode of commuting to school among Spanish youths. Scand J Med Sci Sports. 2018;28(12):2677-85.
46. Potoglou D, Arslangulova B. Factors influencing active travel to primary and secondary schools in Wales. Transportation Planning and Technology. 2017;40(1):80-99.
47. Nelson NM, Woods CB. Neighborhood perceptions and active commuting to school among adolescent boys and girls. Journal of physical activity & health. 2010;7(2):257-66.
48. Larouche R, Sarmiento OL, Broyles ST, Denstel KD, Church TS, Barreira TV, et al. Are the correlates of active school transport context-specific? Int J Obes Suppl. 2015;5(Suppl 2):S89-99.
49. Mathews E, Lakshmi JK, Ravindran TK, Pratt M, Thankappan KR. Perceptions of barriers and facilitators in physical activity participation among women in Thiruvananthapuram City, India. Glob Health Promot. 2016;23(4):27-36.
50. Almahmood M, Scharnhorst E, Carstensen TA, Jørgensen G, Schulze O. Mapping the gendered city: investigating the socio-cultural influence on the practice of walking and the meaning of walkscapes among young Saudi adults in Riyadh. Journal of Urban design. 2017;22(2):229-48.
51. Kroesen M, De Vos J. Does active travel make people healthier, or are healthy people more inclined to travel actively? Journal of transport & health. 2020;16:100844.
52. Ostergaard L, Grontved A, Borrestad LA, Froberg K, Gravesen M, Andersen LB. Cycling to school is associated with lower BMI and lower odds of being overweight or obese

For peer review only - http://bmjopen.bmj.com/site/about/guidelines.xhtml

in a large population-based study of Danish adolescents. Journal of physical activity & health. 2012;9(5):617-25.
53. MacCallum RC, Zhang S, Preacher KJ, Rucker DD. On the practice of dichotomization of quantitative variables. Psychological methods. 2002;7(1):19.
54. World Development Indicators | DataBank: World Development Indicators. Click on a metadata icon for original source information to be used for citation.; 2021 [Available from: https://databank.worldbank.org/reports.aspx?source=2&series=SE.SEC.UNER.LO.ZS&country=#.
55. Air pollution: Asia's deadliest public health crisis isn't COVID. 2021.
56. UNICEF. Child Friendly Cities and Communities Handbook2018.

For peer review only

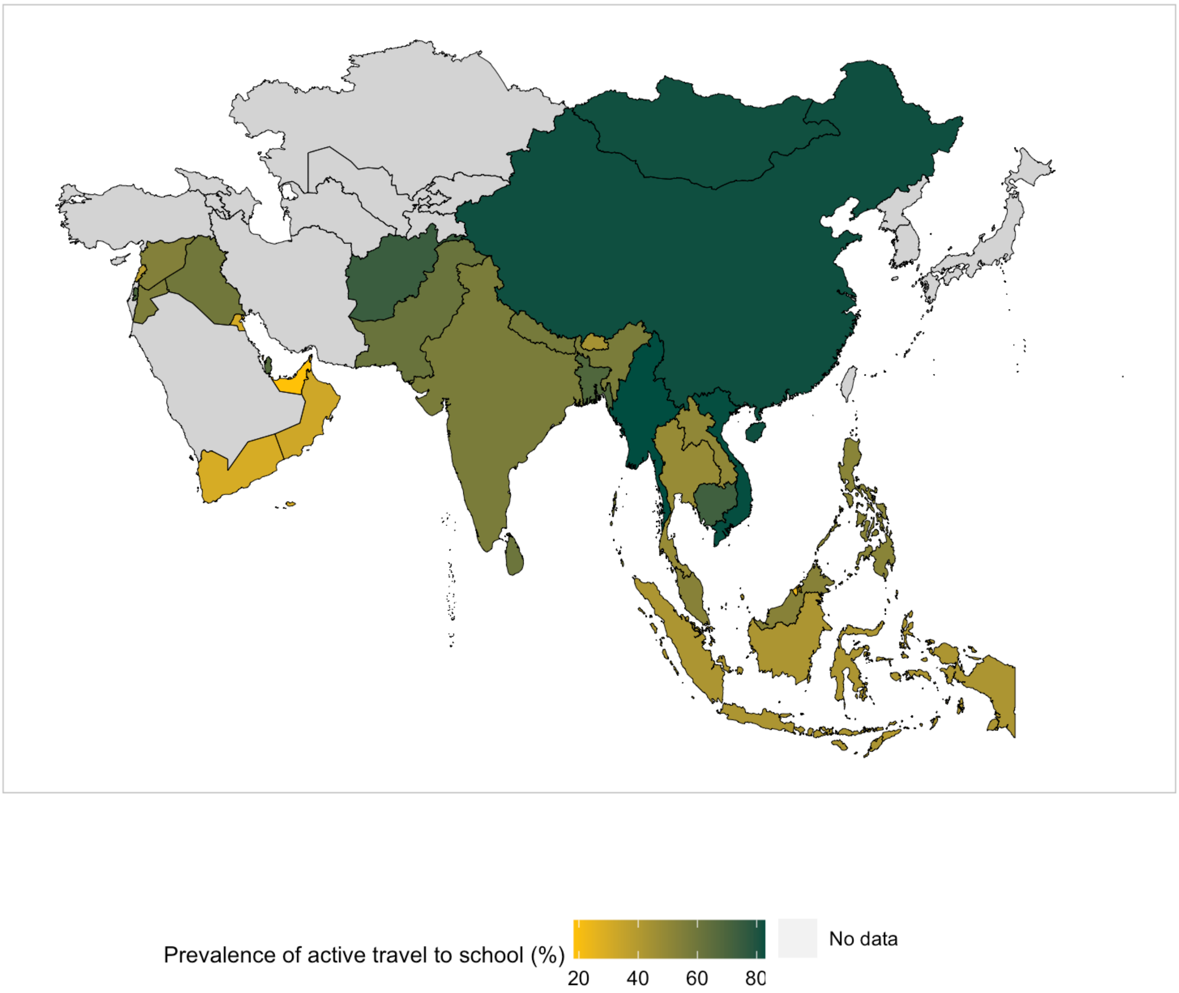
Prevalence of active travel to school (%)
20
40
60
80
No data

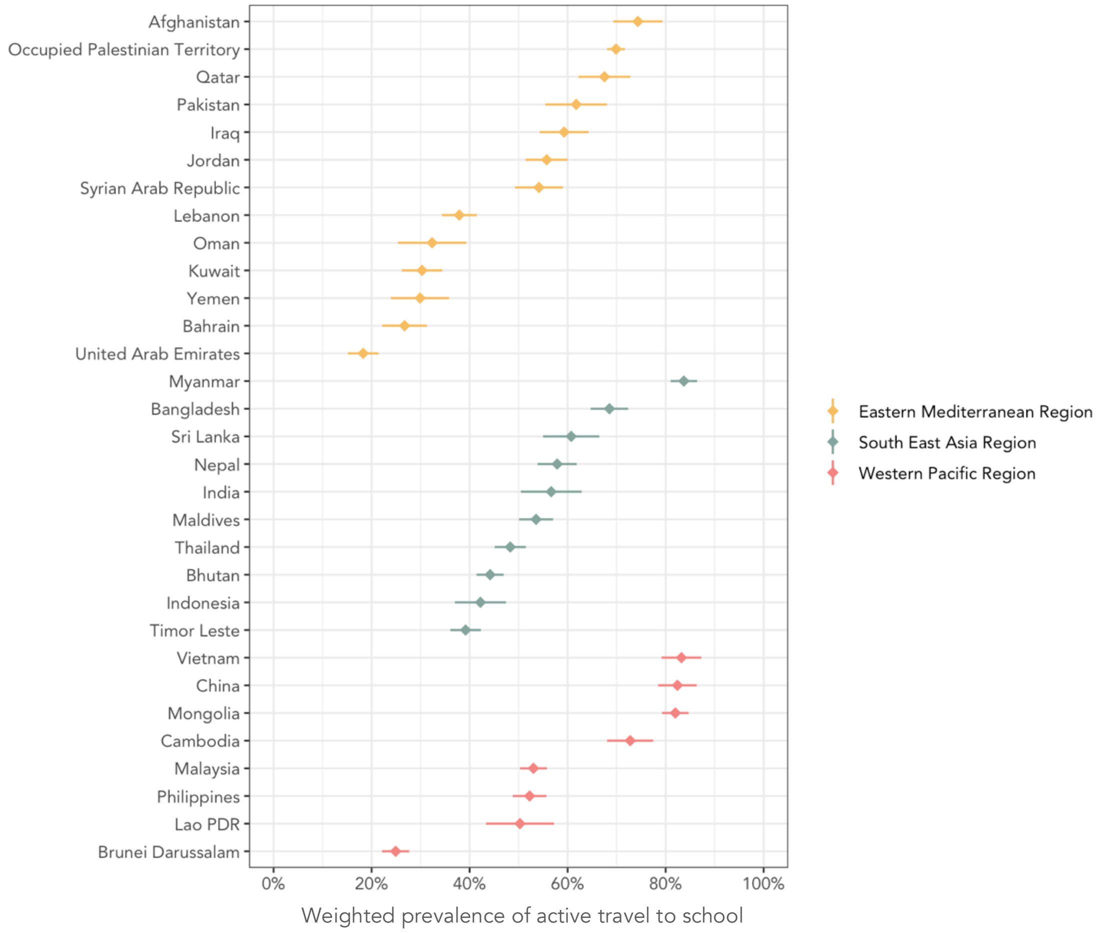
Afghanistan
Occupied Palestinian Territory
Qatar
Pakistan
Iraq
Jordan
Syrian Arab Republic
Lebanon
Oman
Kuwait
Yemen
Bahrain
United Arab Emirates
Myanmar
Bangladesh
Sri Lanka
Nepal
India
Maldives
Thailand
Bhutan
Indonesia
Timor Leste
Vietnam
China
Mongolia
Cambodia
Malaysia
Philippines
Lao PDR
Brunei Darussalam
0%
20%
40%
60%
80%
100%
Weighted prevalence of active travel to school
Eastern Mediterranean Region
South East Asia Region
Western Pacific Region

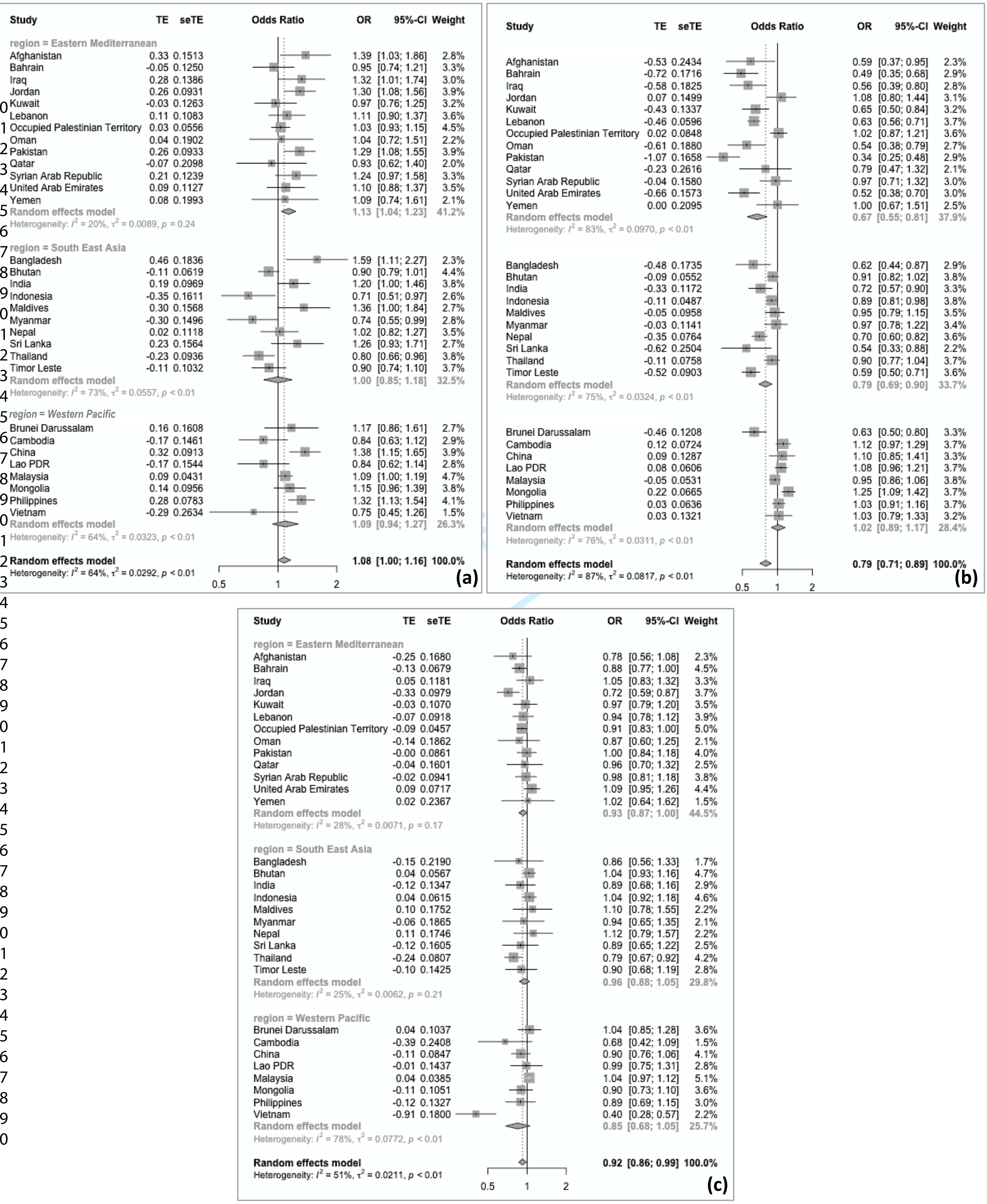
Study TE seTE Odds Ratio OR 95%-CI Weight
region = Eastern Mediterranean
Afghanistan 0.33 0.1513 1.39 [1.03; 1.86] 2.8%
Bahrain -0.05 0.1250 0.95 [0.74; 1.21] 3.3%
Iraq 0.28 0.1386 1.32 [1.01; 1.74] 3.0%
Jordan 0.26 0.0931 1.30 [1.08; 1.56] 3.9%
Kuwait -0.03 0.1263 0.97 [0.76; 1.25] 3.2%
Lebanon 0.11 0.1083 1.11 [0.90; 1.37] 3.6%
Occupied Palestinian Territory 0.03 0.0556 1.03 [0.93; 1.15] 4.5%
Oman 0.04 0.1902 1.04 [0.72; 1.51] 2.2%
Pakistan 0.26 0.0933 1.29 [1.08; 1.55] 3.9%
Qatar -0.07 0.2098 0.93 [0.62; 1.40] 2.0%
Syrian Arab Republic 0.21 0.1239 1.24 [0.97; 1.58] 3.3%
United Arab Emirates 0.09 0.1127 1.10 [0.88; 1.37] 3.5%
Yemen 0.08 0.1993 1.09 [0.74; 1.61] 2.1%
Random effects model 1.13 [1.04; 1.23] 41.2%
Heterogeneity: I^2 = 20%, τ^2 = 0.0089, p = 0.24
region = South East Asia
Bangladesh 0.46 0.1836 1.59 [1.11; 2.27] 2.3%
Bhutan -0.11 0.0619 0.90 [0.79; 1.01] 4.4%
India 0.19 0.0969 1.20 [1.00; 1.46] 3.8%
Indonesia -0.35 0.1611 0.71 [0.51; 0.97] 2.6%
Maldives 0.30 0.1568 1.36 [1.00; 1.84] 2.7%
Myanmar -0.30 0.1496 0.74 [0.55; 0.99] 2.8%
Nepal 0.02 0.1118 1.02 [0.82; 1.27] 3.5%
Sri Lanka 0.23 0.1564 1.26 [0.93; 1.71] 2.7%
Thailand -0.23 0.0936 0.80 [0.66; 0.96] 3.8%
Timor Leste -0.11 0.1032 0.90 [0.74; 1.10] 3.7%
Random effects model 1.00 [0.85; 1.18] 32.5%
Heterogeneity: I^2 = 73%, τ^2 = 0.0557, p < 0.01
region = Western Pacific
Brunei Darussalam 0.16 0.1608 1.17 [0.86; 1.61] 2.7%
Cambodia -0.17 0.1461 0.84 [0.63; 1.12] 2.9%
China 0.32 0.0913 1.38 [1.15; 1.65] 3.9%
Lao PDR -0.17 0.1544 0.84 [0.62; 1.14] 2.8%
Malaysia 0.09 0.0431 1.09 [1.00; 1.19] 4.7%
Mongolia 0.14 0.0956 1.15 [0.96; 1.39] 3.8%
Philippines 0.28 0.0783 1.32 [1.13; 1.54] 4.1%
Vietnam -0.29 0.2634 0.75 [0.45; 1.26] 1.5%
Random effects model 1.09 [0.94; 1.27] 26.3%
Heterogeneity: I^2 = 64%, τ^2 = 0.0323, p < 0.01
Random effects model 1.08 [1.00; 1.16] 100.0%
Heterogeneity: I^2 = 64%, τ^2 = 0.0292, p < 0.01
0.5 1 2
(a)
Study TE seTE Odds Ratio OR 95%-CI Weight
Afghanistan -0.53 0.2434 0.59 [0.37; 0.95] 2.3%
Bahrain -0.72 0.1716 0.49 [0.35; 0.68] 2.9%
Iraq -0.58 0.1825 0.56 [0.39; 0.80] 2.8%
Jordan 0.07 0.1499 1.08 [0.80; 1.44] 3.1%
Kuwait -0.43 0.1337 0.65 [0.50; 0.84] 3.2%
Lebanon -0.46 0.0596 0.63 [0.56; 0.71] 3.7%
Occupied Palestinian Territory 0.02 0.0848 1.02 [0.87; 1.21] 3.6%
Oman -0.61 0.1880 0.54 [0.38; 0.79] 2.7%
Pakistan -1.07 0.1658 0.34 [0.25; 0.48] 2.9%
Qatar -0.23 0.2616 0.79 [0.47; 1.32] 2.1%
Syrian Arab Republic -0.04 0.1580 0.97 [0.71; 1.32] 3.0%
United Arab Emirates -0.66 0.1573 0.52 [0.38; 0.70] 3.0%
Yemen 0.00 0.2095 1.00 [0.67; 1.51] 2.5%
Random effects model 0.67 [0.55; 0.81] 37.9%
Heterogeneity: I^2 = 83%, τ^2 = 0.0970, p < 0.01
Bangladesh -0.48 0.1735 0.62 [0.44; 0.87] 2.9%
Bhutan -0.09 0.0552 0.91 [0.82; 1.02] 3.8%
India -0.33 0.1172 0.72 [0.57; 0.90] 3.3%
Indonesia -0.11 0.0487 0.89 [0.81; 0.98] 3.8%
Maldives -0.05 0.0958 0.95 [0.79; 1.15] 3.5%
Myanmar -0.03 0.1141 0.97 [0.78; 1.22] 3.4%
Nepal -0.35 0.0764 0.70 [0.60; 0.82] 3.6%
Sri Lanka -0.62 0.2504 0.54 [0.33; 0.88] 2.2%
Thailand -0.11 0.0758 0.90 [0.77; 1.04] 3.7%
Timor Leste -0.52 0.0903 0.59 [0.50; 0.71] 3.6%
Random effects model 0.79 [0.69; 0.90] 33.7%
Heterogeneity: I^2 = 75%, τ^2 = 0.0324, p < 0.01
Brunei Darussalam -0.46 0.1208 0.63 [0.50; 0.80] 3.3%
Cambodia 0.12 0.0724 1.12 [0.97; 1.29] 3.7%
China 0.09 0.1287 1.10 [0.85; 1.41] 3.3%
Lao PDR 0.08 0.0606 1.08 [0.96; 1.21] 3.7%
Malaysia -0.05 0.0531 0.95 [0.86; 1.06] 3.8%
Mongolia 0.22 0.0665 1.25 [1.09; 1.42] 3.7%
Philippines 0.03 0.0636 1.03 [0.91; 1.16] 3.7%
Vietnam 0.03 0.1321 1.03 [0.79; 1.33] 3.2%
Random effects model 1.02 [0.89; 1.17] 28.4%
Heterogeneity: I^2 = 76%, τ^2 = 0.0311, p < 0.01
Random effects model 0.79 [0.71; 0.89] 100.0%
Heterogeneity: I^2 = 87%, τ^2 = 0.0817, p < 0.01
0.5 1 2
(b)
Study TE seTE Odds Ratio OR 95%-CI Weight
region = Eastern Mediterranean
Afghanistan -0.25 0.1680 0.78 [0.56; 1.08] 2.3%
Bahrain -0.13 0.0679 0.88 [0.77; 1.00] 4.5%
Iraq 0.05 0.1181 1.05 [0.83; 1.32] 3.3%
Jordan -0.33 0.0979 0.72 [0.59; 0.87] 3.7%
Kuwait -0.03 0.1070 0.97 [0.79; 1.20] 3.5%
Lebanon -0.07 0.0918 0.94 [0.78; 1.12] 3.9%
Occupied Palestinian Territory -0.09 0.0457 0.91 [0.83; 1.00] 5.0%
Oman -0.14 0.1862 0.87 [0.60; 1.25] 2.1%
Pakistan -0.00 0.0861 1.00 [0.84; 1.18] 4.0%
Qatar -0.04 0.1601 0.96 [0.70; 1.32] 2.5%
Syrian Arab Republic -0.02 0.0941 0.98 [0.81; 1.18] 3.8%
United Arab Emirates 0.09 0.0717 1.09 [0.95; 1.26] 4.4%
Yemen 0.02 0.2367 1.02 [0.64; 1.62] 1.5%
Random effects model 0.93 [0.87; 1.00] 44.5%
Heterogeneity: I^2 = 28%, τ^2 = 0.0071, p = 0.17
region = South East Asia
Bangladesh -0.15 0.2190 0.86 [0.56; 1.33] 1.7%
Bhutan 0.04 0.0567 1.04 [0.93; 1.16] 4.7%
India -0.12 0.1347 0.89 [0.68; 1.16] 2.9%
Indonesia 0.04 0.0615 1.04 [0.92; 1.18] 4.6%
Maldives 0.10 0.1752 1.10 [0.78; 1.55] 2.2%
Myanmar -0.06 0.1865 0.94 [0.65; 1.35] 2.1%
Nepal 0.11 0.1746 1.12 [0.79; 1.57] 2.2%
Sri Lanka -0.12 0.1605 0.89 [0.65; 1.22] 2.5%
Thailand -0.24 0.0807 0.79 [0.67; 0.92] 4.2%
Timor Leste -0.10 0.1425 0.90 [0.68; 1.19] 2.8%
Random effects model 0.96 [0.88; 1.05] 29.8%
Heterogeneity: I^2 = 25%, τ^2 = 0.0062, p = 0.21
region = Western Pacific
Brunei Darussalam 0.04 0.1037 1.04 [0.85; 1.28] 3.6%
Cambodia -0.39 0.2408 0.68 [0.42; 1.09] 1.5%
China -0.11 0.0847 0.90 [0.76; 1.06] 4.1%
Lao PDR -0.01 0.1437 0.99 [0.75; 1.31] 2.8%
Malaysia 0.04 0.0385 1.04 [0.97; 1.12] 5.1%
Mongolia -0.11 0.1051 0.90 [0.73; 1.10] 3.6%
Philippines -0.12 0.1327 0.89 [0.69; 1.15] 3.0%
Vietnam -0.91 0.1800 0.40 [0.28; 0.57] 2.2%
Random effects model 0.85 [0.68; 1.05] 25.7%
Heterogeneity: I^2 = 78%, τ^2 = 0.0772, p < 0.01
Random effects model 0.92 [0.86; 0.99] 100.0%
Heterogeneity: I^2 = 51%, τ^2 = 0.0211, p < 0.01
0.5 1 2
(c)

Supplementary

Supplementary Table S1

Table S1. List of countries, year of surveys, and sample sizes from all 31 Asian countries

Country	Year of survey	Sample size
Eastern Mediterranean		
Afghanistan	2014	2028
Bahrain	2016	6928
Iraq	2012	1878
Jordan	2007	1852
Kuwait	2015	2983
Lebanon	2017	4451
Occupied Palestinian territory	2010	12224
Oman	2015	549
Pakistan	2009	4926
Qatar	2011	763
Syrian Arab Republic	2011	2932
United Arab Emirates	2016	5304
Yemen	2008	804
South East Asia		
Bangladesh	2014	2647
Bhutan	2016	7296
India	2007	6524
Indonesia	2015	10423
Maldives and Male	2009	1668
Myanmar	2016	2534
Nepal	2015	5700
Sri Lanka	2016	1618
Thailand	2015	5440
Timor-Leste	2015	2967
Western Pacific		

For peer review only

Brunei Darussalam	2014	2495
Cambodia	2013	3633
China	2003	7679
Lao People’s Democratic Republic	2015	3527
Malaysia	2012	24711
Mongolia	2013	5252
Philippines	2015	7441
Vietnam	2013	3191

For peer review only

Supplementary Figure S1

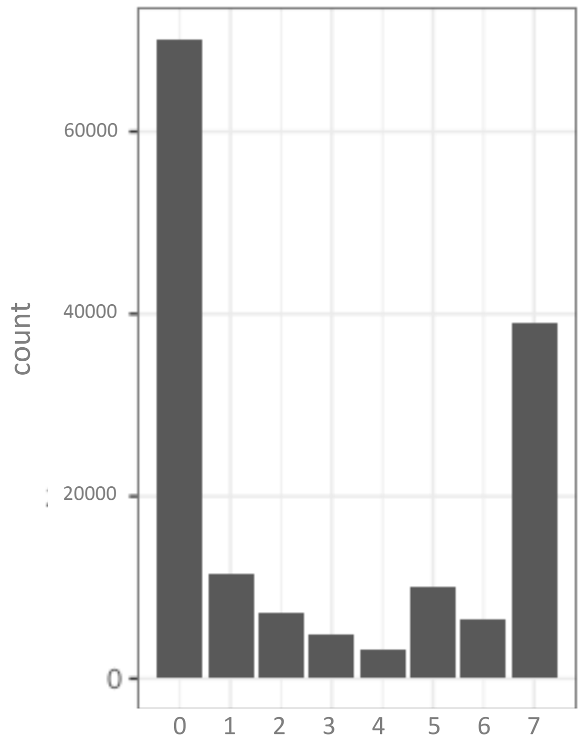

Figure S1. Count distribution of number of days of active travel to school (walking and cycling) in 31 Asian countries

For peer review only

Supplementary Figure S2

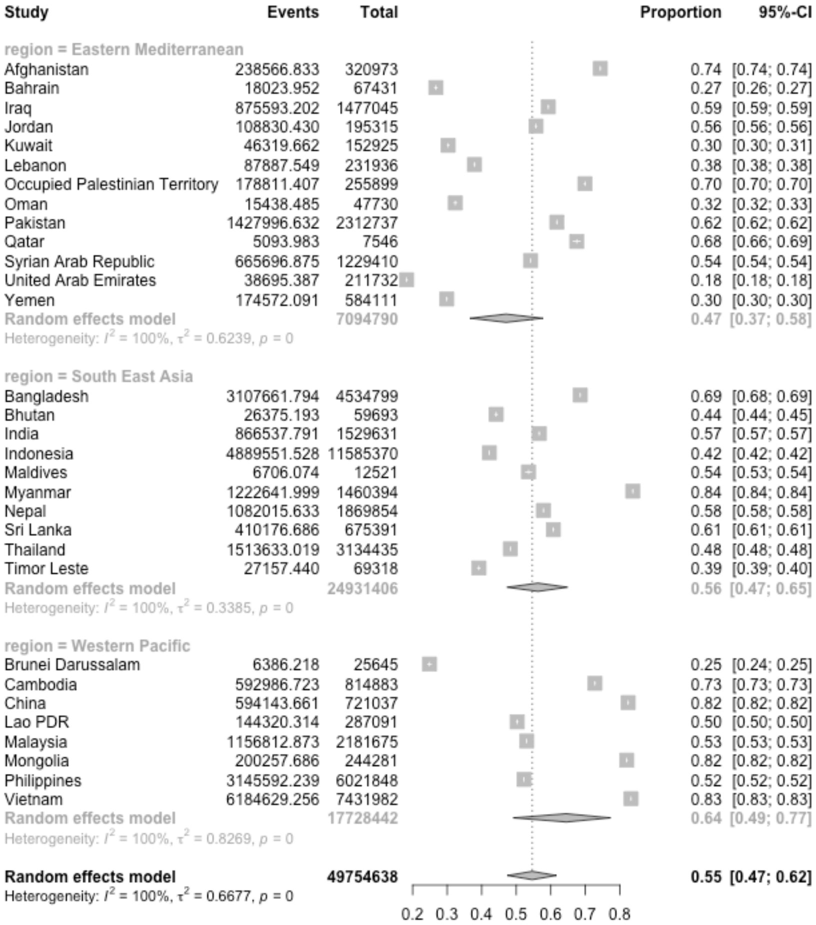

Figure S2. Meta-analysis of prevalence of active travel to school among adolescents in 31 Asian countries

Supplementary Table S2

Table S2. Characteristics comparison between adolescents included in the study, excluded from the study, and in the original dataset (unweighted)

	Complete dataset (n = 174,449)	Observations included in the study (n = 152,368) [a]	Observations with missing data (n = 22,081)	Observations missing weight and height data only (n =19,996)
Age group				
Younger	88,599 (50.8%)	75,655 (49.7%)	12,944 (58.6%)	11,873 (59.4%)
Older	84,957 (48.7%)	76,713 (50.3%)	8,244 (37.3%)	7,230 (36.2%)
NA	893 (0.5%)	-	893 (4.1%)	893 (4.4%)
Sex				
Male	83,090 (47.6%)	72,730 (47.7%)	10,360 (46.9%)	9,291 (46.5%)
Female	89,841 (51.5%)	79,638 (52.3%)	10,203 (46.2%)	9,187 (46.0%)
	1518 (0.9%)	-	1,518 (6.9%)	1,518 (7.5%)
ATS				
Yes	93,034 (53.3%)	82,291 (54.0%)	10,743 (48.7%)	10,743 (53.7%)
No	78,549 (45.0%)	70,077 (46.0%)	8,472 (38.4%)	8,472 (42.4%)
NA	2866 (1.7%)	-	2,866 (12.9%)	781 (3.9%)

[a] Observations with complete ATS, age, sex, weight and height were data included in the analysis.

For peer review only

Supplementary Table S3

Table S3. Meta-regression by year of survey

	**Prevalence**	**Age**	**Sex**	**Weight category**
τ^2 (estimated amount of residual heterogeneity)	0.34	0.023 (SE = 0.01)	0.076 (SE = 0.03)	0.02(SE = 0.01)
τ (square root of estimated tau squared value)	0.58	0.15	0.28	0.13
I^2 (residual heterogeneity / unaccounted variability)	99.99%	61.05%	89.01%	59.97%
H^2 (unaccounted variability / sampling variability)	89240.43	2.57	9.10	2.50
R^2 (amount of heterogeneity accounted for)	N/A	21.77%	7.13%	15.19%
Tests for residual heterogeneity	Wld (df = 19) = 762219.74 (p-value < .0001) LRT (df = 19) = 865578.68 (p-value < .0001)	QE (df = 19) = 43.62 (p-value = 0.01)	QE (df = 19) = 126.65 (p-value < .0001)	QE (df = 19) = 34.83 (p-value = 0.02)
Test of moderators (coefficients 2:12)	QM (df = 11) = 30.03 (p-value = 0.01)	QM (df = 11) = 15.12 (p-value = 0.18)	QM (df = 11) = 12.61 (p-value = 0.32)	QM (df = 11) = 11.36 (p-value = 0.41)

Df = data frame; LRT = likelihood ratio test statistic of the test for (residual) heterogeneity; QE = test statistic for the test of (residual) heterogeneity; QM = test statistic of the omnibus test of moderators; Wld = Wald-type test statistic of the test for residual heterogeneity

For peer review only

STROBE Statement—Checklist of items that should be included in reports of ***cross-sectional studies***

	Item No	Recommendation	Page No
**Title and abstract**	1	(*a*) Indicate the study's design with a commonly used term in the title or the abstract	1
		(*b*) Provide in the abstract an informative and balanced summary of what was done and what was found	2
**Introduction**			
Background/rationale	2	Explain the scientific background and rationale for the investigation being reported	3
Objectives	3	State specific objectives, including any prespecified hypotheses	4
**Methods**			
Study design	4	Present key elements of study design early in the paper	5
Setting	5	Describe the setting, locations, and relevant dates, including periods of recruitment, exposure, follow-up, and data collection	5
Participants	6	(*a*) Give the eligibility criteria, and the sources and methods of selection of participants	5-6
Variables	7	Clearly define all outcomes, exposures, predictors, potential confounders, and effect modifiers. Give diagnostic criteria, if applicable	6
Data sources/ measurement	8*	For each variable of interest, give sources of data and details of methods of assessment (measurement). Describe comparability of assessment methods if there is more than one group	6
Bias	9	Describe any efforts to address potential sources of bias	7
Study size	10	Explain how the study size was arrived at	5
Quantitative variables	11	Explain how quantitative variables were handled in the analyses. If applicable, describe which groupings were chosen and why	6
Statistical methods	12	(*a*) Describe all statistical methods, including those used to control for confounding	6
		(*b*) Describe any methods used to examine subgroups and interactions	7
		(*c*) Explain how missing data were addressed	6
		(*d*) If applicable, describe analytical methods taking account of sampling strategy	7
		(*e*) Describe any sensitivity analyses	7
**Results**			
Participants	13*	(a) Report numbers of individuals at each stage of study—eg numbers potentially eligible, examined for eligibility, confirmed eligible, included in the study, completing follow-up, and analysed	8
		(b) Give reasons for non-participation at each stage	8
		(c) Consider use of a flow diagram	N/A
Descriptive data	14*	(a) Give characteristics of study participants (eg demographic, clinical, social) and information on exposures and potential confounders	8
		(b) Indicate number of participants with missing data for each variable of interest	8
Outcome data	15*	Report numbers of outcome events or summary measures	8
Main results	16	(*a*) Give unadjusted estimates and, if applicable, confounder-adjusted estimates and their precision (eg, 95% confidence interval). Make clear which confounders were adjusted for and why they were included	8-9

For peer review only - http://bmjopen.bmj.com/site/about/guidelines.xhtml

		(*b*) Report category boundaries when continuous variables were categorized	8
		(*c*) If relevant, consider translating estimates of relative risk into absolute risk for a meaningful time period	N/A
Other analyses	17	Report other analyses done—eg analyses of subgroups and interactions, and sensitivity analyses	9
**Discussion**			
Key results	18	Summarise key results with reference to study objectives	10
Limitations	19	Discuss limitations of the study, taking into account sources of potential bias or imprecision. Discuss both direction and magnitude of any potential bias	12
Interpretation	20	Give a cautious overall interpretation of results considering objectives, limitations, multiplicity of analyses, results from similar studies, and other relevant evidence	10-12
Generalisability	21	Discuss the generalisability (external validity) of the study results	12
**Other information**			
Funding	22	Give the source of funding and the role of the funders for the present study and, if applicable, for the original study on which the present article is based	14

*Give information separately for exposed and unexposed groups.

Note: An Explanation and Elaboration article discusses each checklist item and gives methodological background and published examples of transparent reporting. The STROBE checklist is best used in conjunction with this article (freely available on the Web sites of PLoS Medicine at http://www.plosmedicine.org/, Annals of Internal Medicine at http://www.annals.org/, and Epidemiology at http://www.epidem.com/). Information on the STROBE Initiative is available at www.strobe-statement.org.

For peer review only
